# Thermoresponsive Graft Copolymers of *N*‑Isopropylacrylamide and Hyperbranched Polyglycerol as Thermally Induced Drug Delivery and Release Nanoformulation Systems for Curcumin with High Colloidal Stability and Enhanced Anticancer Effect

**DOI:** 10.1021/acsomega.5c05838

**Published:** 2026-01-08

**Authors:** György Kasza, Ákos Fábián, Dóra Fecske, Anna Petróczy, Kata Horváti, Béla Iván

**Affiliations:** † Polymer Chemistry and Physics Research Group, Institute of Materials and Environmental Chemistry, 280964 HUN-REN Research Centre for Natural Sciences, Magyar Tudósok Körútja 2, H-1117 Budapest, Hungary; ‡ Hevesy György Doctoral School of Chemistry, ELTE Eötvös Loránd University, Pázmány Péter sétány 1/A, H-1117 Budapest, Hungary; § MTA-HUN-REN TTK ″Momentum″ Peptide-Based Vaccines Research Group, Institute of Materials and Environmental Chemistry, HUN-REN Research Centre for Natural Sciences, Magyar Tudósok Körútja 2, H-1117 Budapest, Hungary

## Abstract

The poor solubility, stability issues, and restricted bioavailability of numerous drugs and potential pharmaceutical compounds highlight the critical need for the development of polymeric nanoparticles as effective drug delivery systems. Ideal polymers for such applications must be biocompatible, provide controlled drug loading and release, and maintain the high colloidal stability of the nanoformulation. In this study, LCST-type thermoresponsive poly­(*N*-isopropylacrylamide)-*g*-(hyperbranched polyglycerol) (PNiPAAm-*g*-HbPG) graft copolymers, composed of two biocompatible components, were synthesized by grafting amino-monofunctional HbPG, prepared by multibranching anionic ring-opening polymerization, onto PNiPAAm chains containing succinimide active ester groups. In both aqueous solutions and phosphate-buffered saline, these graft copolymers undergo reversible aggregation and disaggregation in response to temperature changes, with linear increase of the critical solution temperature (CST) as a function of grafting density. The architecture of the copolymers enables efficient encapsulation of curcumin, a natural anticancer agent, by a simple mixing process at elevated temperatures, and the hydrophilic HbPG side chains provide excellent colloidal stability for the polymer–drug nanoformulations. Sustained release of curcumin from the PNiPAAm-*g*-HbPG aggregates was found, which depends on the grafting density, and the release rate can be externally triggered by decreasing the temperature. The potential biocompatibility of the PNiPAAm-*g*-HbPG graft copolymers was demonstrated *in vitro*, with no evidence of cytotoxicity or hemolytic activity against human cells. The nanoformulations of curcumin with these new graft copolymers enhanced its internalization into HT-29 human colorectal adenocarcinoma cells and significantly improved its cytostatic effect against colon cancer cells. These properties of the novel PNiPAAm-*g*-HbPG graft copolymers make them superior candidates for advanced drug encapsulation and delivery systems.

## Graphical abstract

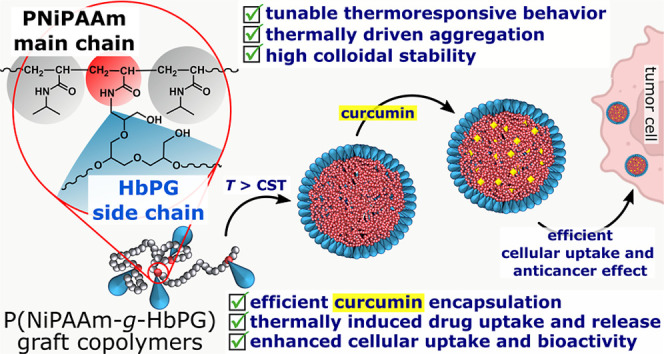

## Introduction

Polymer-based delivery systems are undoubtedly one of the most extensively investigated classes of materials for drug formulations. Considering their ability to adjust the concentration of the drug in the body by increasing its solubility, controlling the rate of drug release, and specifically targeting the site of action, they can positively influence the bioactivity, bioavailability, biodistribution, and stability of the drugs. Over the years, various polymer-based drug delivery systems have been investigated, including well-defined functionalized polymers for conjugation with drugs and targeting moieties^1–3^ and especially amphiphilic polymers that exhibit self-assembling properties to form nanocarriers (e.g., micelles,^4–7^ nanoparticles,^8–11^ polymersomes,^12,13^ etc.) for drug encapsulation in a noncovalent manner.^14^ Among such macromolecular assemblies, responsive (smart, intelligent, adaptive) polymers have gained notable interest in drug formulation due to the abrupt changes in their physical or chemical properties caused by external stimuli, which can trigger drug uptake and release.^15–18^ These include polymers responding to changes in the temperature, light, electromagnetic fields, pH, or ultrasound. Nevertheless, thermoresponsive polymers have been the most extensively investigated in this field due to the ease of stimuli by heating or cooling.^19–22^ The thermoresponsive nature of polymer solutions is characterized by LCST or UCST phenomena, corresponding to the temperatures at which the critical solution temperature (CST) reaches its minimum or maximum, respectively, in the polymer/solvent phase diagram.^23,24^ Several (co)­polymers exhibit thermoresponsive behavior,^25,26^ such as poly­(2-alkyl-2-oxazoline)­s,^7,27–32^ various poly­(meth)­acrylamides,^33–36^ oligo- and poly­(ethylene glycol)-(meth)­acrylate-based polymers,^37–45^ poly­(methyl vinyl ether),^46^ poly­(*N,N*-dimethylaminoethyl methacrylate),^47–51^ and so on. However, poly­(*N*-isopropylacrylamide) (PNiPAAm) is still the most widely studied thermoresponsive polymer.^52–54^ The CST of an aqueous solution of PNiPAAm is close to that of the human body, at approximately 32–34 °C, allowing its use in biomedical applications, particularly in drug delivery. At and above the CST, PNiPAAm chains undergo a reversible coil-to-globule (hydrated-dehydrated) transition and simultaneous aggregation to form colloidal suspensions or macroscopic precipitates.^55,56^ To enhance the colloidal stability and to tune the thermoresponsive and drug delivery properties, PNiPAAm-based copolymers, including block,^57–61^ random,^58,59^ and graft copolymers,^58–60,62^ have been intensively investigated.

Poly­(ethylene glycol)­s (PEGs) are biocompatible materials with prolonged blood circulation time, and therefore, copolymerization of PNiPAAm with PEGs is commonly implemented to adjust the CST.^20,63^ Above the CST, PEG-containing copolymers demonstrate enhanced colloidal stability.^64,65^ Comparatively, PNiPAAm copolymers with PEG side chains show a more pronounced effect on CST than those with PEG incorporated as a block.^43,64,66^ Although PEG is one of the most extensively investigated synthetic polymers for bioapplications, it has several known disadvantages,^67–69^ such as possible accumulation in the body, hypersensitivity, formation of anti-PEG antibodies, accelerated blood clearance, and nonbiodegradability, indicating an increasing need for PEG alternatives with more advantageous properties than that of PEG.

According to our concept, hyperbranched polyglycerol (HbPG), which possesses numerous advantageous properties, including biocompatibility, excellent water solubility, and high colloidal stability,^70–72^ can be applied as a PEG alternative to obtain hydrophilic side-chain-grafted thermoresponsive copolymers. Additionally, the biomedical application possibilities of HbPG are being widely investigated in several directions, such as drug delivery, inhibition of viral infections, and antifouling coatings.^73,74^ Recently, thermoresponsive acrylated HbPG-functionalized β-cyclodextrin was copolymerized with NiPAAm leading to gels with enhanced topical drug delivery,^75^ indicating the advantage of the presence of HbPG in such biomedically viable macromolecular constructs. A variety of hypergrafted copolymers of HbPG have also been studied, in which the HbPG was grafted onto linear polymer backbones^76–81^ or thermoresponsive polymers were grafted onto the HbPG core^82–84^ to obtain copolymers with beneficial characteristics. Biocompatible HbPG-grafted poly­(*N*,*N*-diethylacrylamide) (PDEAAm) thermoresponsive copolymers, indicating the advantages of HbPG grafts in such macromolecular assemblies, have been recently reported by us.^85^

This study deals with PNiPAAm-*g*-HbPG graft copolymers with composition-dependent thermoresponsive behavior as novel carriers with high colloidal stability and *in vitro* susceptibility for drug encapsulation and release of cargo with low water solubility. The targeted PNiPAAm-*g*-HbPG-s were obtained by utilizing a well-defined amine monofunctional HbPG^86^ for highly efficient postpolymerization grafting onto copolymers of NiPAAm with *N*-acryloxysuccinimide (NAOS), as a reactive comonomer, which has already been applied to obtain PNiPAAm-*g*-PEG brush copolymers.^87^ Subsequently, thermally induced encapsulation and release, cell internalization, and anticancer effect of curcumin, a natural antioxidant, anti-inflammatory, antibacterial, and anticancer agent,^88–92^ with the thermoresponsive PNiPAAm-*g*-HbPG graft copolymers as drug carrier nanoformulations were investigated and are reported herein.

## Experimental Section

### Materials

Glycidol (from Sigma-Aldrich) was freshly distilled under reduced pressure before use. *N*-isopropylacrylamide (NiPAAm from Sigma-Aldrich) was purified by recrystallization in *n*-hexane. Azobis­(isobutyronitrile) (AIBN, Sigma-Aldrich) was recrystallized in methanol two times before use. The following reagents were utilized directly as supplied: acrylic acid *N*-hydroxysuccinimide ester (*N*-acryloxysuccinimide, NAOS, >90%, Sigma-Aldrich), phthalimide (≥99%, Sigma-Aldrich), potassium phthalimide (≥99%, Sigma-Aldrich), hydrazine monohydrate (reagent grade, ≥97%, Sigma-Aldrich), phosphate-buffered saline (PBS) tablets (Sigma-Aldrich), Tween 80 (Sigma-Aldrich), lithium bromide (99.99%, Alfa Aesar), and curcumin (97%, Apollo Scientific). Solvents including methanol (MeOH), ethanol (EtOH), diethyl ether, and *n*-hexane (all from Molar Chemicals Ltd.), as well as *N*,*N*-dimethylformamide (DMF, HPLC grade, VWR Chemicals) were employed without further purification. Tetrahydrofuran (THF, ≥99.5%, Molar Chemicals Ltd.) was refluxed over potassium hydroxide and distilled prior to use as the polymerization medium or applied directly for precipitation. For the *in vitro* assays, RPMI-1640 medium, PBS (pH = 7.4), fetal bovine serum (FBS), l-glutamine, trypsin, and penicillin–streptomycin were purchased from Thermo Fisher Scientific (Gibco; Waltham, MA, USA). AlamarBlue reagent was from Merck (Budapest, Hungary).

### Synthesis Methods

#### Synthesis of Thermoresponsive Poly­(*N*-isopropylacrylamide) (PNiPAAm) Homopolymer and Poly­(*N*-isopropylacrylamide)-*co*-(*N*-acryloxysuccinimide) (P­(NiPAAm-*co*-NAOS)) Copolymers

Free radical polymerization was employed to synthesize the PNiPAAm homopolymer as well as the P­(NiPAAm-*co*-NAOS) copolymers, following the procedure described recently by us.^85^ The initiator/monomer molar ratio was 1:100 in all cases, and the NiPAAm/NAOS comonomer ratios were 20:1, 10:1, and 5:1. As an example, the synthesis of P­(NiPAAm-*co*-NAOS) with a NiPAAm/NAOS ratio of 20:1 is described below. NiPAAm (1.9002 g, 16.8 mmol) and NAOS (0.1420 g, 0.84 mmol) were dissolved in 16 mL of distilled THF, and the resulting solution was heated to 60 °C under nitrogen. Subsequently, 2.76 mL of AIBN in a THF stock solution (10 mg/mL) was introduced to the reaction mixture, and it was stirred under a nitrogen atmosphere at 60 °C for 16 h. After cooling, the reaction mixture was poured in hexane, and the resulting precipitate was isolated by centrifugation. The polymer was then dried under vacuum at 60 °C until a constant mass was obtained. The yields of the P­(NiPAAm-*co*-NAOS) copolymers were >90% in all cases.

#### Synthesis of Amine-Monofunctional HbPG (NH_2_–HbPG)

The NH_2_–HbPG was synthesized by a two-step method.^86^ Briefly, phthalimide monofunctional HbPG (PhthIm-HbPG) was produced by phthalimide/potassium phthalimide-initiated ring-opening multibranching polymerization of glycidol. Phthalimide (1.4999 g, 10.2 mmol) and potassium phthalimide (0.2154 g, 1.2 mmol) were measured into a three-necked round-bottomed flask equipped with a mechanical stirrer and connected to a vacuum line, while the third neck was sealed with a septum. The reaction flask was heated to 95 °C and was purged with a stream of nitrogen. Subsequently, 9 mL (10.065 g, 136 mmol) of glycidol was added dropwise (flow rate: 2.5 mL/h) to the reaction flask via a syringe pump. The reaction mixture was stirred for an additional 18 h to ensure complete incorporation of the monomer. The crude product was cooled and dissolved in ethanol, passed through a column filled with a cation exchange resin, and precipitated in diethyl ether. It was then dried under a vacuum at 60 °C (yield = 89%). Subsequently, 1.45 mL of hydrazine monohydrate (1.5043 g, 30.1 mmol) was added to the PhthIm-HbPG (*M*_n_ = 1180 g/mol determined by ^1^H NMR, see Figure S1, 1.6527 g, 1.5 mmol) dissolved in 20 mL of EtOH and the reaction mixture was stirred overnight at room temperature. After filtration, the crude product was obtained by precipitation in a large excess of a THF/diethyl ether mixture (2:1 *V*/*V*) followed by centrifugation. The precipitation step was performed twice to remove residual impurities, after which the amine-functional HbPG was dried under vacuum at 45 °C (yield: 75%; *M*_n_ = 1050 g/mol from ^1^H NMR; Figure S2). The gel permeation chromatography (GPC) chromatograms and MWD curves of the phthalimide- and amine-monofunctional HbPGs are presented in Figure S3.

#### Synthesis of PNiPAAm-*g*-HbPG Copolymers by Grafting of P­(NiPAAm-*co*-NAOS) Copolymers with the NH_2_–HbPG

The grafting of the HbPG onto the NAOS-containing PNiPAAm copolymers was performed according to our recently presented method.^85^ In a typical grafting procedure, 100 mg of P­(NiPAAm-*co*-NAOS) was dissolved in MeOH, and a methanolic solution containing NH_2_–HbPG in an equimolar amount relative to the NAOS units was added. After the polymer concentration was adjusted to 10 mg/mL, the solution was stirred at ambient temperature overnight. Thereafter, the products were purified by dialysis (SpectraPor 6, MWCO 1 kDa) against distilled water for 3 days to remove NHS byproduct and unreacted HbPG and then lyophilized.

### Characterizations

#### Gel Permeation Chromatography

GPC analyses were performed on a setup consisting of a Waters 515 pump and a JASCO AS-4150 autosampler, with Styragel HR1, HR4, and HR6 columns connected in series and a JASCO 4035 refractive-index detector. The eluent was DMF containing LiBr of 1 g/L concentration with a flow rate of 0.3 mL/min at 50 °C. The average molecular weights and dispersity values were obtained by calibration using PEG standards (PSS, 194–217,000 Da).

#### NMR Spectroscopy

^1^H NMR analyses were performed on a Varian Inova spectrometer at a 500 MHz resonance frequency. The polymers were analyzed in deuterated dimethyl sulfoxide (DMSO-*d*_6_) and deuterium oxide (D_2_O) at 30 °C.

#### Thermoresponsive Behavior Investigations

Turbidimetric analyses were conducted to evaluate the thermoresponsive behavior of the homopolymer and copolymer samples. Measurements were performed on a JASCO V-650 spectrophotometer equipped with a temperature-control system (JASCO MCB-100 circulation unit and a Peltier thermostat). For the NAOS-containing copolymers, transmittance was recorded at 1 g/L in water, while the HbPG-grafted derivatives were examined in both water and PBS at polymer concentrations of 1, 5, and 10 g/L. All experiments were carried out at 488 nm wavelength over a temperature range of 20–80 °C using a heating/cooling rate of 0.5 °C min^–1^, with a 1 min equilibration at each temperature step. The cloud point (*T*_CP_) and clearing point (*T*_CL_) were determined as the inflection points of the corresponding transmittance–temperature curves during the heating and cooling cycles, respectively.^23,24^

#### Encapsulation of Curcumin

A stock solution of curcumin (0.5 mL, 20 g/L in acetone) was added dropwise to 1 mL of an aqueous solution of PNiPAAm-*g*-HbPG with varying polymer concentrations (from 0.1 to 10 mg/mL in distilled water under vigorous stirring at 25 and 37 °C). Subsequently, the stirring was continued for an additional 4 h to permit the evaporation of the acetone. The polymer concentration was maintained by the addition of water, if necessary. The suspensions were filtered through a 0.45 μm syringe filter to remove the insolubilized curcumin. Subsequently, 50 μL of the filtered solution was taken and diluted with 0.95 mL of ethanol, and the solution was analyzed by spectrophotometry (JASCO V-650) at 25 °C. The encapsulated drug contents were evaluated by a curcumin calibration curve by the absorbance at 424 nm in absolute EtOH, i.e., at a wavelength having no absorbance for the PNiPAAm-*g*-HbPG graft copolymers (see Figures S14 and S15). All the measurements were performed in triplicate. The encapsulation was expressed in the drug loading content (*DLC*), which was determined by the following equation

1 $$DLC=\frac{encapsulated\;curcumin\;concentration}{encapsulated\;curcumin\;concentration+polymer\;concentration}$$

Statistical analysis was performed using a two-sample *t*-test, statistical significance:

*p < 0.05, ***p < 0.001. All data are presented as the mean ± SD, *N* = 3.

#### Colloidal Stability

The colloidal stability of the PNiPAAm homopolymer, the PNiPAAm-*g*-HbPG graft copolymers, and curcumin in both native and nanoformulated forms was analyzed in PBS by using a JASCO V-650 spectrophotometer equipped with a JASCO MCB-100 circulation unit and a Peltier-based temperature controller. Polymer concentrations were set at 10 g/L for the homopolymer and copolymers, while the drug-containing nanoformulations were prepared at 5 g/L with the maximum curcumin loading. After 10 min of equilibration at 37 °C under constant stirring at 600 rpm, stirring was stopped, and the transmittance at 600 nm was monitored over 24 h with PBS serving as the reference medium.

#### Dynamic Light Scattering Measurements

The hydrodynamic diameter and the size distribution of the PNiPAAm-*g*-HbPG copolymers and the curcumin-loaded nanoformulations were investigated by dynamic light scattering (DLS) using a Zetasizer Nano ZS (Malvern Instruments, Malvern, UK) with an operating wavelength of 633 nm and a measurement angle of 173°. Measurements of the pure graft copolymers were performed at a polymer concentration of 1 g/L in water without filtration. Before each measurement at varying temperatures, the samples were equilibrated for 30 min. The curcumin-loaded nanoparticles were prepared by the same method described in the previous section, using a polymer concentration of 1 g/L in PBS and a 100 μM curcumin concentration. Subsequently, the samples were filtered through a 0.45 μm membrane filter and subsequently measured at 37 °C after 30 min of equilibration time. The particle size distribution of the samples was determined using statistical data analysis methods (cumulant analysis and the CONTIN algorithm) of the resulting autocorrelation data. The data presented in this paper represent the mean distributions calculated from three independent replicates.

#### Drug Release

The release of curcumin from the PNiPAAm-*g*-HbPG nanoformulations prepared at 25 and 37 °C was examined using the well-established dialysis method^85,110,111^ and compared to the release of free curcumin (1 g/L curcumin in DMSO/water = 40/60 *V*/*V* %). Immediately after preparation, 1 mL of each curcumin-loaded nanoformulation (*c*_polymer_ = 5 mg/mL) was transferred into a glass tube fitted with 3.5 kDa SpectraPor 6 dialysis membranes and the other end was sealed with a cap. The tubes were then immersed in 40 mL of PBS containing 3% Tween 80, stirred at 150 rpm, and maintained at 37 ± 0.5 °C. At predetermined intervals, 50 μL of the samples were withdrawn and diluted with 0.95 mL of EtOH, and the drug content was quantified spectrophotometrically (424 nm, 25 °C, JASCO V-650). An additional experiment was carried out similarly, with the exception that the temperature of the medium was reduced to 32 °C after 1 h. All the measurements were conducted in triplicate.

The effect of the temperature reduction on the curcumin content of the graft copolymer-based formulations was investigated. After preparing the drug-loaded aggregates at 37 °C, the solutions were cooled to 32 and 25 °C. Any precipitated curcumin was separated via filtering with a 0.45 μm syringe filter. The concentration of curcumin was then determined by using a JASCO V-650 spectrophotometer at 424 nm and ambient temperature. All measurements were carried out in triplicate.

#### Hemolysis Assay

The hemolytic activity of the polymers was tested on human red blood cells (RBCs), obtained from healthy volunteers, by the Hungarian National Blood Transfusion Service (Budapest, Hungary). First, RBCs were washed twice with PBS, then diluted with PBS (2% *V*/*V*), and plated on a 96-well, round-bottom plate (Sarstedt, Nümbrecht, Germany) at a volume of 50 μL. Solutions were prepared at a volume of 50 μL with concentrations of 1.25, 2.5, and 5 g/L and added to the erythrocytes. The final RBC concentration was 1% *V*/*V*, and the final volume was 100 μL in the wells. Plates were incubated at 37 °C, 5% CO_2_ for 2 h, centrifuged at 2000 rpm (5 min, 4 °C), and then 50 μL of the supernatants were carefully transferred to a new plate containing 100 μL of H_2_O in each well. Optical density was measured at 414 and 450 nm by a Synergy 2 multimode microplate reader (BioTek, Winooski, VT, USA), and the percentage of hemolysis compared to a positive control (bee venom Melittin, 10 μM, in PBS) was determined. All data are presented as the mean ± SD, *N* = 3.

#### Cytostasis Assay on HT29 Cells

First, viability of HT-29 colorectal adenocarcinoma cells was tested after treatment with the polymers at 2, 0.67, and 0.22 g/L concentrations. Cells were seeded 1 day prior to the experiment in a 96-well tissue culture plate (Sarstedt) at a density of 10,000 cells in 100 μL per well in RPMI-1640 medium. The day after, cells were treated with polymers for 24 h (37 °C in 5% CO_2_), then washed 3 times, and incubated for a further 2 days in 10% FBS-containing RPMI-1640 medium. Percentage of viability, compared to medium-treated cells, was measured by using the (4,5-dimethylthiazol-2-yl)-2,5-diphenyltetrazolium bromide (MTT) colorimetric assay described by Mossmann^106^ and modified by Horváti.^107^ All data are presented as the mean ± SD, *N* = 4.

The cytostatic effect of curcumin and curcumin-loaded polymers was tested, as described above. To avoid possible precipitation of free curcumin, which could hamper the readability of the assay, the final curcumin concentration was adjusted to 7 μM in all formulations, including both free and encapsulated samples, while the polymer concentration was 0.67 g/L.

Statistical analysis was performed using one-way analysis of variance (ANOVA), followed by Tukey’s post hoc test. Statistical significances were denoted as ***p < 0.0001.

#### Internalization Study

Internalization of the curcumin and curcumin-loaded polymers was measured in the HT-29 cells. One day before the experiment, cells were seeded in a 24-well tissue culture plate (Sarstedt, 100,000 cells/well in 1 mL of RPMI-1640 medium). The next day, cells were treated with 5 μM curcumin, with or without the polymers. The polymer concentration was 0.5 g/L. After incubation for 3 h, cells were washed with serum-free RPMI-1640 medium. Then, the supernatant was removed, and 100 μL of 0.25% trypsin was added to the wells. After 10 min of incubation, 0.8 mL of 10% FBS/RPMI-1640 medium was added and then transferred to 5 mL FACS tubes. Cells were washed two times with PBS, and then the intracellular fluorescence intensity of the cells was measured on a CytoFLEX (Beckman Coulter, Brea, California, US) on an FITC fluorescence channel (excitation λ = 488 nm, emission at λ = 525/40 nm). All measurements were performed in triplicate, and the mean fluorescent intensity together with SD was presented. Statistical analysis was performed using one-way ANOVA, followed by Tukey’s post hoc test. Statistical significance was denoted as ***p < 0.001.

## Results and Discussion

### The Synthesis of P­(NiPAAm-*co*-NAOS) and the PNiPAAm-*g*-HbPG Copolymers

Scheme 1 shows the synthetic route of the preparation of PNiPAAm-*g*-HbPG graft copolymers. First, NiPAAm-based copolymers containing reactive NAOS comonomer units were prepared via free radical copolymerization using three distinct comonomer ratios. The obtained comonomer ratios in the resulting P­(NiPAAm-*co*-NAOS) copolymers were evaluated from the integral ratios of the methine protons of the NiPAAm isopropyl groups and the methylene protons of NAOS (2.88–3.13 ppm) in the ^1^H NMR spectra (Figure S1). It was found that the amount of NAOS incorporated was slightly less than that of the feed ratio. Specifically, the molar contents of NAOS in the copolymers are 4.3, 7.8, and 15.7 mol % for samples with 4.8, 9.1, and 16.7 mol % NAOS in the feed, respectively. Afterward, the measured comonomer contents, that is, 4.3, 7.8, and 15.7, will be used for sample identification. The amino-monofunctional HbPG (NH_2_–HbPG, *M*_n,NMR_ = 1050 g/mol, PDI∼1.2; see Figures S2–S4) was obtained via a two-step procedure described in our prior work.^86^ Specifically, monofunctional phthalimide-HbPG was synthesized by anionic ring-opening multibranching polymerization of glycidol, employing a phthalimide/phthalimideK initiator system, followed by hydrazinolysis to obtain the amino-functional polymer, as illustrated in Scheme 1, step 2. Subsequently, the obtained amino-HbPG was grafted onto the P­(NiPAAm-*co*-NAOS) precursors in MeOH at room temperature, as shown in Scheme 1, step 3.

**Scheme 1:**
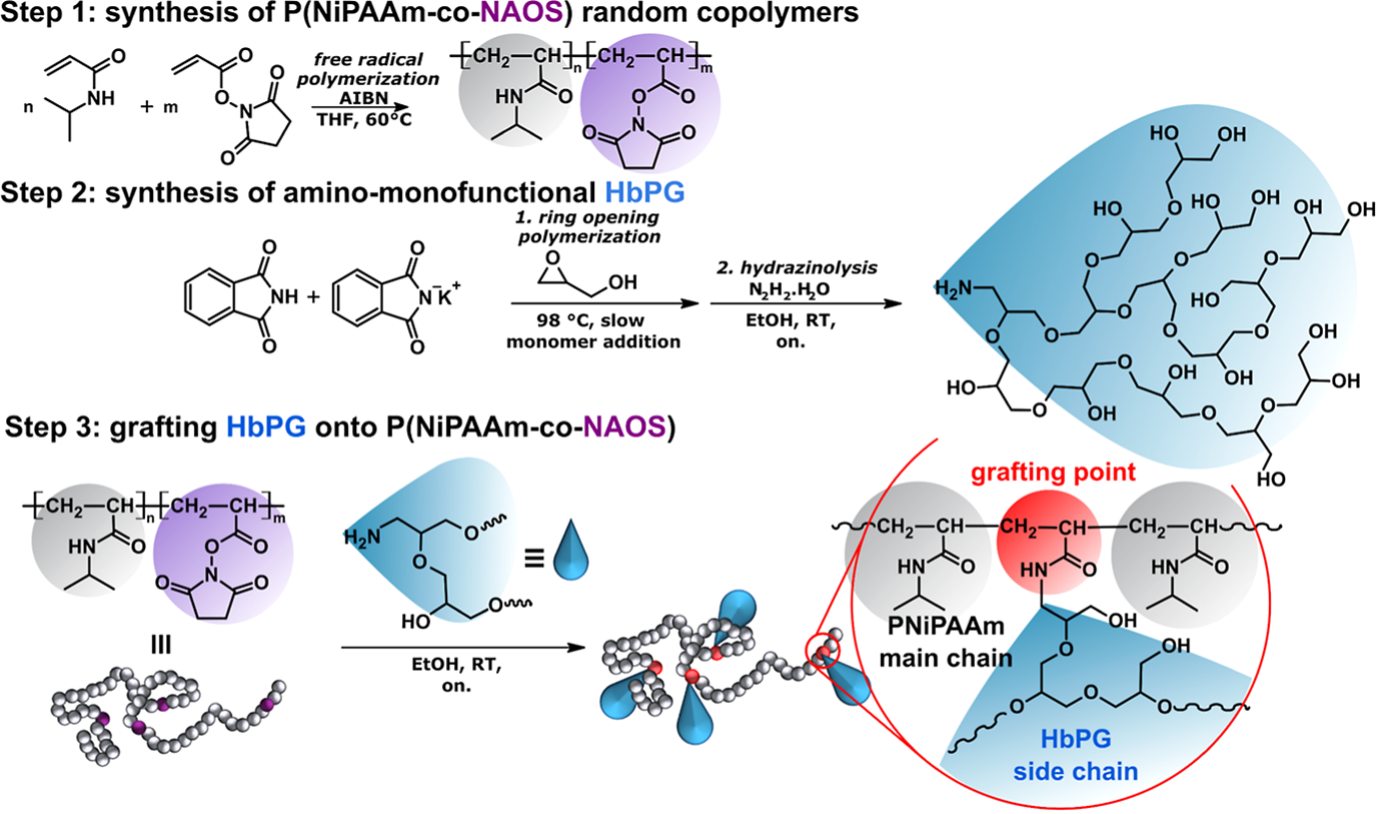
Synthetic Approach for Poly­(*N*-isopropylacrylamide)-*g*-(HbPG) (PNiPAAm-*g*-HbPG) Graft Copolymers

Based on the GPC curves (Figure S5), the P­(NIPAAm-*co*-NAOS) copolymers are formed within a similar molecular weight range with a PDI of approximately 2. It has to be noted that the average molecular weights are relative values due to the differences in the hydrodynamic volumes of the linear homopolymer standards used to calibrate GPC and those of the prepared copolymers. Comparing the GPC curves of the PNiPAAm-*g*-HbPG graft copolymers to those of the initial P­(NiPAAm-*co*-NAOS) copolymers indicates that the relative amount of the small molecular weight fraction decreases. At the same time, the curves broaden, and a shoulder appears in the high molecular weight range for all graft copolymers, suggesting the successful grafting of the HbPG. The appearance of the shoulder in the higher molecular weight fraction could be explained by the different solvation, thereby the different hydrodynamic properties of this fraction containing more hydrophilic HbPG. The conjugation reaction between the P­(NiPAAm-*co*-NAOS) and the NH_2_–HbPG is also supported by the ^1^H NMR spectroscopy results (see Figure S1). The characteristic signal of the succinimidyl functional groups of NAOS in the 2.8–3.1 ppm range does not appear in the spectra of the PNiPAAm-*g*-HbPG products, and the appearance of the broad peaks of the methylene and methine protons next to the ether groups in HbPG in the range of 3.4–4.1 ppm confirms the presence of HbPG in the resulting polymers, i.e., the formation of PNiPAAm-*g*-HbPG graft copolymers by grafting of HbPG onto the P­(NiPAAm-*co*-NAOS) copolymers. In this work, the term grafting density refers to the number of HbPG chains attached per 100 monomer units, which is directly determined from the mol % NAOS content obtained by ^1^H NMR analysis.

### The Thermoresponsive Behavior of the P­(NiPAAm-*co*-NAOS) and the PNiPAAm-*g*-HbPG Copolymers

The thermoresponsive properties of both the P­(NiPAAm-*co*-NAOS) copolymers and the PNiPAAm-*g*-HbPG graft copolymers were studied by turbidimetric measurements. In our study, turbidity is defined and measured via transmittance at 488 nm using a UV–Vis spectrophotometer. The recorded Tr-*T* curves of the P­(NiPAAm-*co*-NAOS) copolymer precursors and the HbPG-grafted PNiPAAm-based copolymers in water are presented in Figure 1A,B, respectively (for their first derivative curves, see Figure S6). The turbidimetry results of the PNiPAAm homopolymer are displayed in Figure S7. The determined *T*_CP_ and *T*_CL_ values for all of the investigated polymers are listed in Table S1.

**Figure 1:**
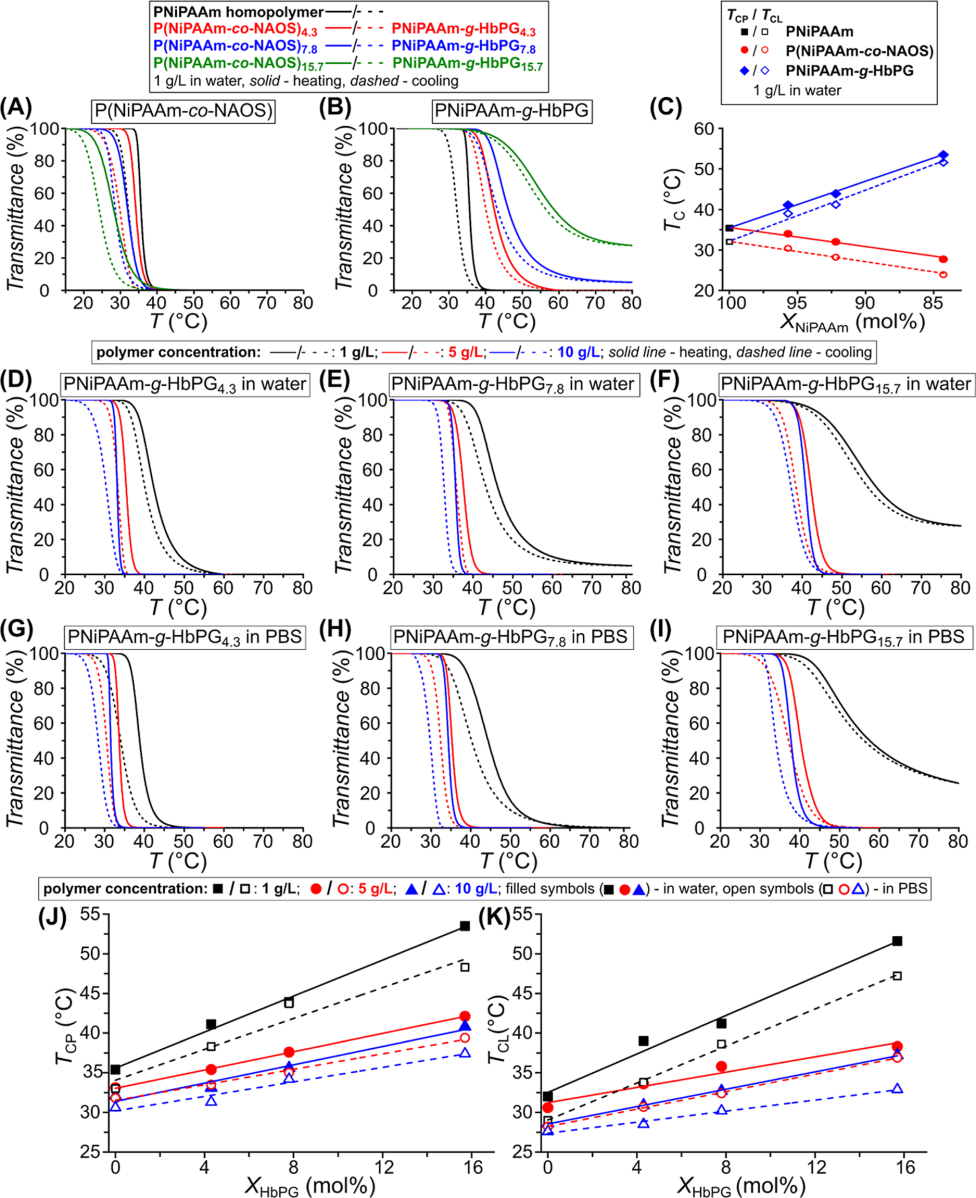
Transmittance vs temperature curves of the PNiPAAm homopolymer and the P­(NiPAAm-*co*-NAOS) copolymers (A) and the PNiPAAm-*g*-HbPG copolymers (B) during heating (solid line) and cooling (dashed line) at a 1 g/L concentration in water. *T*_CP_ (filled symbols) and *T*_CL_ (open symbols) values as a function of the NiPAAm molar fraction at a 1 g/L polymer in water (C). Transmittance vs temperature curves of the PNiPAAm homopolymer and the PNiPAAm-*g*-HbPG copolymers at different concentrations (1, 5, 10 g/L) during heating (solid line) and cooling (dashed line) in water (D–F) and in PBS buffer (G–I). The *T*_CP_ and the *T*_CL_ values as a function of the HbPG molar content (G,H) in water (filled symbols) and in PBS (open symbols) at different concentrations. Lines in panels C, J, and K are added to guide the eye. (The transmittance was measured at 488 nm, and the numbers in sample codes indicate the NAOS comonomer contents or HbPG contents in mol %.)

Figure 1A shows that the transmittance of the P­(NiPAAm-*co*-NAOS) copolymers and PNiPAAm decreases to zero within a narrow temperature range and exhibits reversibility in heating–cooling cycles. It has to be noted that despite the fact that the synthesis and application of P­(NiPAAm-*co*-NAOS) copolymers have already been reported,^87,93–96^ the impact of the NAOS comonomer on the thermoresponsive behavior has not been investigated so far. As shown in Figure 1C, the values of both the *T*_CP_ and the *T*_CL_ decrease linearly with increasing NAOS content in the P­(NiPAAm-*co*-NAOS) copolymers, which is a consequence of the hydrophobic nature of the NAOS comonomer, in line with the effect of other hydrophobic comonomers.^97^

This finding indicates that the CST values decrease by approximately 0.5 °C/mol % as the mole fraction of NAOS increases. As can be seen in Figure 1A,C, there is a notable hysteresis between the *T*_CP_ and *T*_CL_ values for the PNiPAAm homopolymer^23^ and also in the case of the P­(NiPAAm-*co*-NAOS) copolymers due to the well-known fact that PNiPAAm forms intermolecular and intramolecular hydrogen bonds in the precipitated stage above the CST. However, this hysteresis is not observed for the P­(DEAAm-*co*-NAOS) copolymers, which are free from hydrogen bonding as reported recently.^85^ Nevertheless, the increase of the NAOS content does not lead to a decrease in hysteresis, which suggests that this monomer is also involved in the formation of secondary interactions, i.e., hydrogen bonding with NiPAAm monomer units, and consequently, responsible also for the appearance of hysteresis. The CST values were also determined for the PNiPAAm-*g*-HbPG graft copolymers at a 1 g/L polymer concentration (Figure 1B). Contrary to the P­(NiPAAm-*co*-NAOS) copolymers, a linear increase in CST values was observed with increasing the HbPG content of the copolymers as shown in Figure 1C. This result unambiguously demonstrates the successful grafting of HbPG to the PNiPAAm-*co*-NAOS copolymers. The molar effect of HbPG on the CST value is more pronounced than that of the NAOS. Specifically, the CST value of the PNiPAAm-*g*-HbPG copolymers increases approximately by 1.15 °C/mol % of grafting side chains, which is attributed to the high hydrophilicity of the HbPG chains. This trend is in good agreement with our previous observations on PDEAAm-*g*-HbPG copolymers, where an analogous increase in CST with increasing HbPG content was also detected.^85^ Surprisingly, the hysteresis between the *T*_CP_ and *T*_CL_ values measured for the PNiPAAm-*g*-HbPG copolymers is smaller than that for the P­(NiPAAm-*co*-NAOS) copolymers, indicating that the bulky hydrophilic HbPG grafts suppress hydrogen bonding between the PNiPAAm chain segments to some extent.

The Tr-*T* curves of the PNiPAAm homopolymer and PNiPAAm-*g*-HbPG copolymer solutions were measured at varying polymer concentrations (1, 5, 10 g/L) in water and a physiological PBS buffer, as shown in Figure 1D–I. The transmittance change is less sharp in the case of the PNiPAAm-*g*-HbPG graft copolymers than that for the PNiPAAm homopolymer at the lowest polymer concentration (1 g/L), occurring within a range from 40 to 60 °C, from 45 to 70 °C, and from 45 to 80 °C for the PNiPAAm-*g*-HbPG_4.3_, PNiPAAm-*g*-HbPG_7.8,_ and PNiPAAm-*g*-HbPG_15.7_ samples, respectively. The corresponding *T*_CP_ and *T*_CL_ values are plotted against the grafting density, i.e., the molar fraction of the HbPG in the copolymers, and against the polymer concentration, as shown in Figure 1J,K. The data shown indicate that both the cloud point (*T*_CP_) and clearing point (*T*_CL_) decrease with increasing polymer concentration, independent of copolymer composition or the solvent used.^85^ Notably, at the same polymer concentration, the transmittance changes for the PNiPAAm-*g*-HbPG copolymers appear at higher temperatures compared with the homopolymer. This trend demonstrates that both *T*_CP_ and *T*_CL_ rise substantially with increasing HbPG graft density in water and PBS (Figure 1J,K). As expected, the presence of salt, i.e., the use of PBS, has a notable impact on the thermoresponsive behavior. Namely, similar to the recently reported PDEAAm-*g*-HbPG copolymers,^85^ the CST values in PBS are about 2–3 °C lower than in water, although there is a slight decline in this effect with increasing polymer concentration. When using PNiPAAm-*g*-HbPG graft copolymer-based drug delivery systems, it is important to consider that increasing the HbPG content while decreasing the polymer concentration may limit the ability to exploit the thermoresponsive nature, as the CST values of graft copolymers with more HbPG at lower concentrations in PBS exceed body temperature.

The thermoresponsive behavior of the graft copolymers was also investigated by DLS measurements. Figure 2A–C presents the size distribution of the PNiPAAm-*g*-HbPG graft copolymers with a 1 g/L polymer concentration at varying temperatures. As observed, the graft copolymers exhibit a mean diameter of approximately 5–10 nm at room temperature, which increases markedly around *T*_CP_ and levels off over 50 °C in the 350–550 nm size range. Upon returning to room temperature, the mean diameter exhibits the same small values observed at the initial stage of the heating process, indicating the reversible nature of the thermally induced transition. Figure 2D–F shows the hydrodynamic diameter values as a function of the temperature, overlaid with the turbidimetric curves measured at the same concentration. Although the initial temperature of the size change is often used in the literature to characterize the CST,^98^ the coil-to-globule and globule-to-aggregate transitions take place simultaneously, i.e., at this temperature, the aggregation of partially dehydrated chains can commence instantaneously before the entire polymer undergoes the coil-to-globule transition.

**Figure 2:**
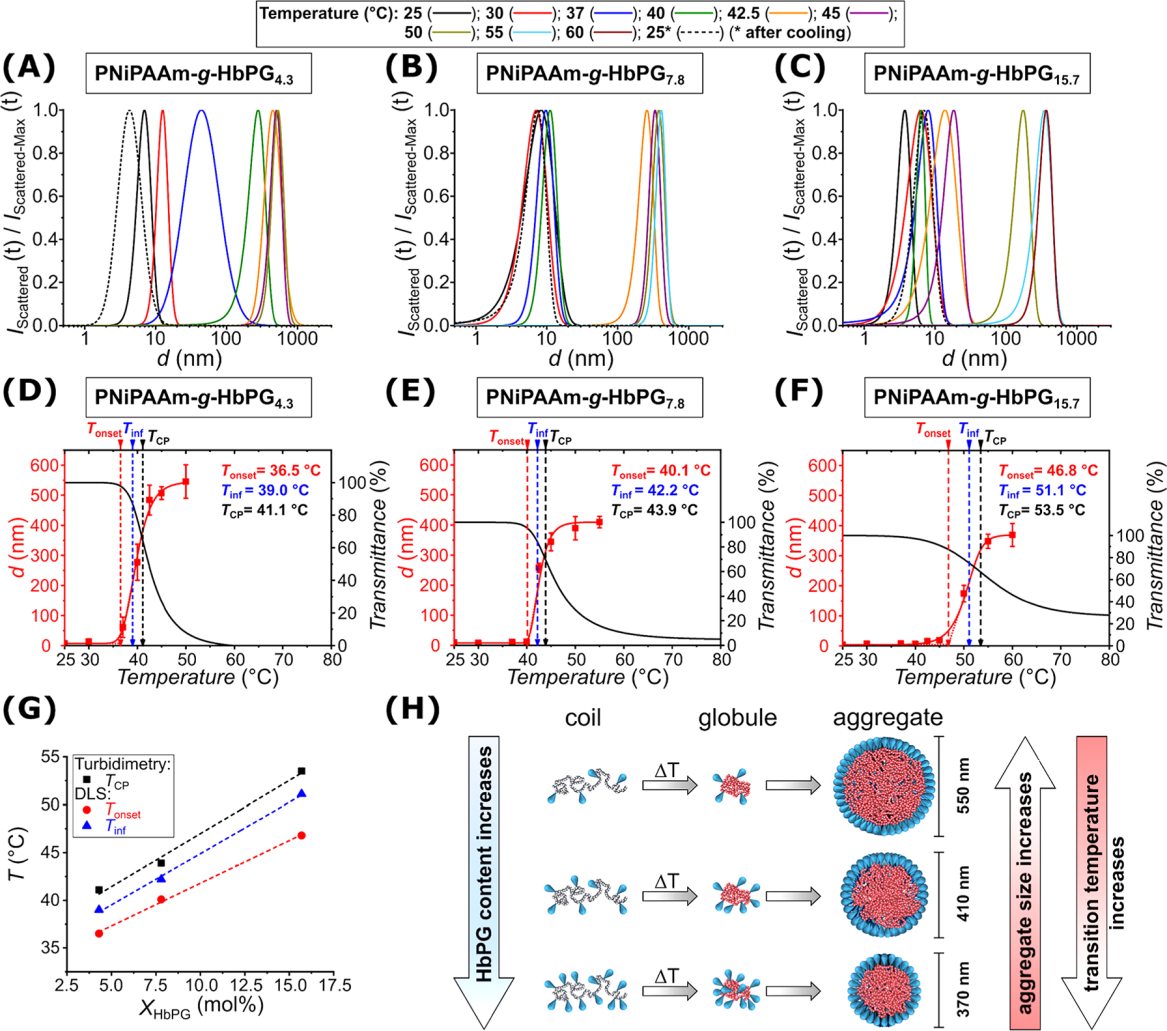
Size distribution curves (A–C) of the PNiPAAm-*g*-HbPG graft copolymers at different temperatures during heating (solid lines) and after cooling back to 25 °C (dashed line) determined by DLS measurements. Temperature dependence of the mean hydrodynamic diameter (*d*, red symbols and lines) during a heating cycle for the PNiPAAm-*g*-HbPG graft copolymers and the transmittance versus temperature curves (black lines) measured by turbidimetry (D–F). Comparison of the onset temperatures (*T*_onset_, red), the inflection temperature (*T*_inf_, blue) of size increase, and the *T*_CP_ values (black) determined by turbidimetry measurements (G). Schematic illustration of the composition-dependent coil-to-globule-to-aggregate transition of the PNiPAAm-*g*-HbPG graft copolymers (H). (The numbers in sample codes indicate the HbPG contents in mol %.)

Consequently, similar to the turbidimetric studies, the temperature of the coil-to-globule-to-aggregate transition can be identified as the temperature at the inflection point of the sigmoid curve fitted to the size versus temperature data. The temperatures at the inflection points (*T*_inf_) are consistent with the CST values determined by turbidimetric measurement and exhibit a similar trend as displayed in Figure 2G. The *T*_inf_ increases with increasing HbPG graft density, and the difference between these values and the *T*_CP_ determined by turbidimetry is about 1.7–2.4 °C. This discrepancy between these values can be attributed to the sensitivity of DLS and/or the differences in the applied concentrations, heating and cooling rates, and the wavelength used in turbidimetry and DLS measurements.^23,99^ As presented in Figure 2D–F, in addition to *T*_inf_, the initial temperature of size change (*T*_onset_) and the maximum mean diameter are also affected by the composition of the PNiPAAm-*g*-HbPG graft copolymers. Increasing the HbPG content results in higher onset temperatures and smaller maximum average sizes of the aggregates above the CST, i.e., 550, 410, and 370 nm for copolymers with 4.3, 7.8, and 15.7 mol % of HbPG, respectively. This indicates hindered aggregation by the hydrophilic HbPG side chains leading to smaller aggregates above the CST, which is in accordance with the turbidimetric results. At the same concentration, the transmittance of the copolymer solutions with a higher HbPG content does not reach zero even at 80 °C. Specifically, the transmittance values were approximately 8% and 35% for the PNiPAAm-*g*-HbPG_7.8_ and PNiPAAm-*g*-HbPG_15.7_ samples, respectively. These findings indicate that in addition to the CST, the extent of copolymer aggregation, which leads to the formation of nanoparticles with PNiPAAm-enriched hydrophobic core and a well-hydrated HbPG shell, can be regulated by the HbPG content of the copolymers, i.e., the grafting density, as depicted in Figure 2H.

### Drug Delivery Properties of the PNiPAAm-*g*-HbPG Graft Copolymers

The results presented in the previous section on the thermoresponsive behavior of PNiPAAm-*g*-HbPG graft copolymers have revealed that these copolymers form nanosized aggregates composed of hydrophobic PNiPAAm and hydrophilic HbPG segments at specified concentrations above their CST. Consequently, the amphiphilic nature of these nanoassemblies may enable the encapsulation of hydrophobic drugs. Therefore, the drug delivery properties of the graft copolymers were investigated by determining the drug uptake and release profile of curcumin.

The amount of curcumin encapsulated within PNiPAAm-*g*-HbPG aqueous solutions was measured by UV–vis spectroscopy across a range of polymer concentrations at 25 and 37 °C (Figure 3A,B). Figure 3 shows that increasing the polymer concentration results in an increase in the concentration of encapsulated curcumin. At higher polymer concentrations, the concentration of curcumin using the PNiPAAm-*g*-HbPG copolymers is 2 or 3 orders of magnitude higher than its reported water solubility of 0.6 mg/L at room temperature.^100,101^ Importantly, the control experiment performed with pure HbPG (10 g/L) showed no detectable increase in the curcumin concentration. At higher PNiPAAm-*g*-HbPG concentrations (*c*_polymer_ > 2 g/L), the concentration of the encapsulated curcumin increases even at room temperature, i.e., below the CST of the graft copolymers. This finding suggests a moderate interaction between the hydrophobic curcumin and NiPAAm segments of the graft copolymers, while the HbPG-enriched hydrophilic outer shell remains intact.

**Figure 3:**
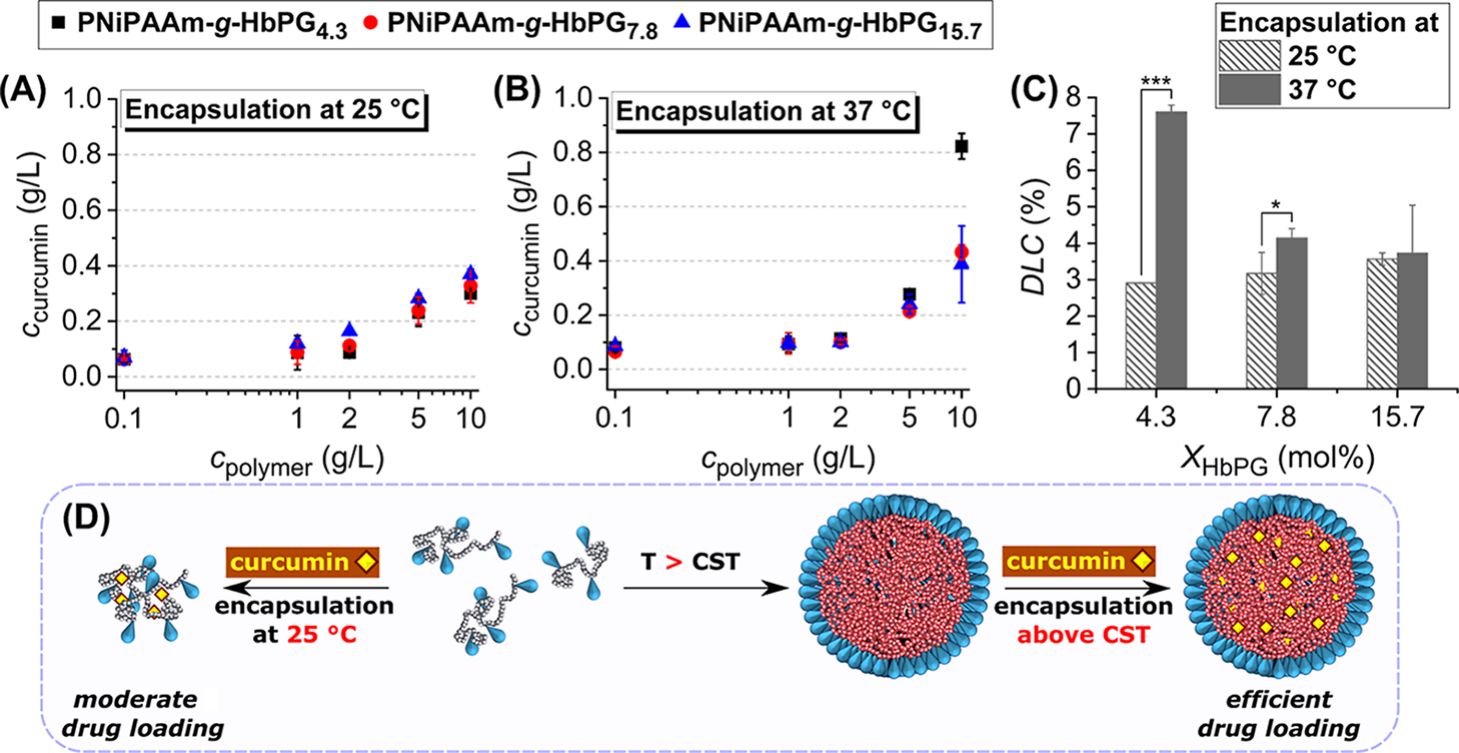
Concentration of the encapsulated curcumin by the PNiPAAm-*g*-HbPG graft copolymers at 25 °C (A) and 37 °C (B) plotted against the concentration of the formulating polymers. Curcumin DLC in the nanoformulations as a function of the HbPG molar content of the PNiPAAm-*g*-HbPG graft copolymers at 25 °C (striped) and 37 °C (filled) at 10 g/L of PNiPAAm-*g*-HbPG graft copolymer concentration (C) (data represent mean ± Sd, *N* = 3; statistical analysis was performed using a two-sample *t*-test, statistical significance: *p < 0.05, ***p < 0.001). Schematic illustration of the drug loading at 25 °C and above the CST (D). (The numbers in sample codes indicate the HbPG contents in mol %.)

Figure 3C shows that the composition has a considerable influence on the encapsulation capabilities, as evidenced by the comparison of the drug loading content (DLC) at a polymer concentration of 10 g/L. As obtained, while there is no significant difference in the DLC at room temperature, drug loading contents are consistently higher at 37 °C, and the increasing HbPG content reduces the relative encapsulated drug content. This finding is related to the relative hydrophobic nature of the copolymer aggregates; that is, increasing the HbPG content of the copolymer increases the CST values and decreases the apparent hydrophobicity of the PNiPAAm segments at 37 °C, resulting in a reduction of the encapsulation capacity. Consequently, a higher degree of curcumin encapsulation can be achieved at elevated temperatures and a higher NiPAAm content of the graft copolymer nanocarrier. As recently reported, the PDEAAm-*g*-HbPG copolymers exhibit higher curcumin encapsulation even at room temperature due to the more hydrophobic nature of PDEAAm compared to that of PNiPAAm.^85^ Therefore, the temperature-dependent drug uptake property of the PNiPAAm-based graft copolymers, which is illustrated in Figure 3D, suggests the possibility of a thermally induced release profile, in contrast to the PDEAAm-containing analogs for which the absence of this ability was obtained.^85^

The drug release profile of curcumin from the polymer aggregates (*c*_polymer_ = 5 mg/mL), and also the free form as control to demonstrate the applicability of the utilized measurement technique (1 g/L curcumin in DMSO/water = 40/60 V/V %), was investigated by using the dialysis method in PBS (pH = 7.4) in the presence of 3% Tween 80 at 37 °C.^85,110,111^ The drug release is expressed as the cumulative release as a function of the release time (Figure 4A). The measured curcumin concentration as a function of the release time can be seen in Figure S9. As shown, free curcumin in DMSO/water exhibits rapid diffusion through the dialysis membrane, resulting in over 90% release within approximately 5 h. However, an increase in the HbPG content in the PNiPAAm-*g*-HbPG graft copolymers results in a slower sustained release of curcumin. This effect can be attributed to the fact that a higher HbPG content results in a denser and well-hydrated surface region of the particles, which suppresses the transfer of hydrophobic curcumin to the medium. A similar trend was observed in the case of PDEAAm-*g*-HbPG graft copolymer, as reported recently.^85^ These findings are also consistent with those of Wu et al.,^102^ who demonstrated that high-molecular-weight HbPG delays the release of hydrophobic guest molecules.

**Figure 4:**
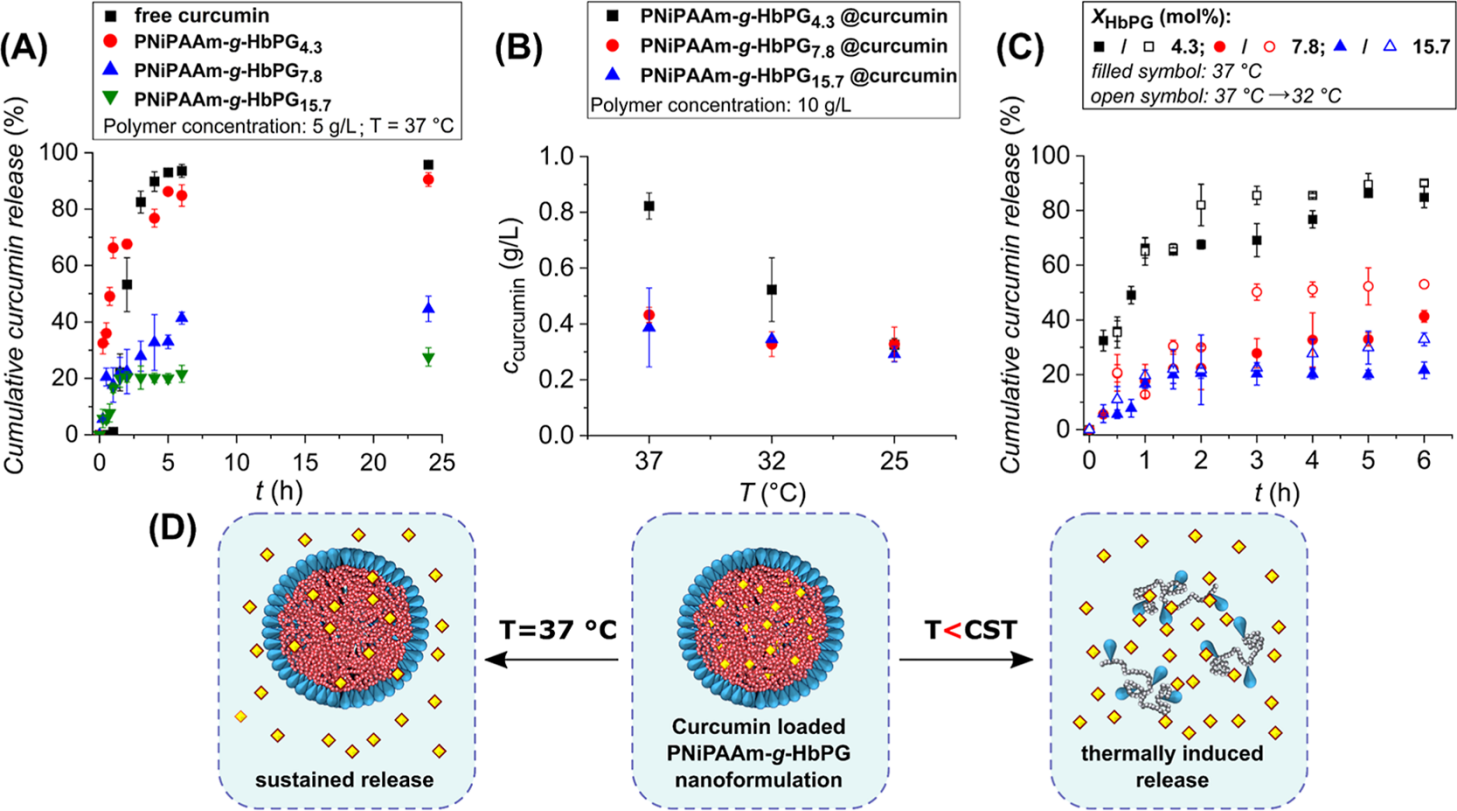
Time-dependent cumulative release profiles of curcumin from the PNiPAAm-*g*-HbPG graft copolymers compared with free curcumin (A). Encapsulated curcumin concentration at 37 °C and after subsequent cooling to 32 and 25 °C (B). Temperature-dependent cumulative release profiles of encapsulated curcumin from the PNiPAAm-*g*-HbPG graft copolymers, where the release medium was maintained at 37 °C (filled symbols) and cooled to 32 °C after 1 h (open symbols) (C). Schematic illustration of the drug release of curcumin (yellow dots) from the NiPAAm-*g*-HbPG graft copolymers at constant 37 °C and at a reduced temperature below the CST (D) (data represent mean ± Sd, *N* = 3, and the numbers in sample codes indicate the HbPG contents in mol %.)

One of the most significant advantages of thermoresponsive polymers is their ability to facilitate the controlled release of drugs in response to changes in the temperature. Consequently, the effect of cooling on the curcumin content and release properties was investigated. The concentration of the encapsulated curcumin was evaluated at 37 °C and after cooling to 32 and 25 °C (Figure 4B), revealing a notable decrease in drug concentration as the temperature is lowered.

The data in Figure 4B also indicate that increasing the HbPG grafting density results in a less pronounced loss of the encapsulated curcumin content upon cooling. This finding aligns with the encapsulation results; i.e., the lower HbPG content of the graft copolymers leads to enhanced encapsulation ability at elevated temperatures, and following cooling, the encapsulated surplus of curcumin is fully released. The release is less temperature-dependent when the HbPG content is higher, due to the fact that the CST of the copolymer containing the highest amount of HbPG at the applied polymer concentration (5 g/L) is above 37 °C. Nevertheless, these findings clearly prove that cooling the environment can trigger drug release from the PNiPAAm-*g*-HbPG graft copolymers. Consequently, to reveal the impact of modifying the temperature of the medium on the release profile, drug release experiments were conducted with a reduction in temperature from 37 to 32 °C after 1 h. Figure 4C displays the initial release of curcumin during the first 6 h, while the subsequent decrease in curcumin concentration and the cumulative release over 24 h are depicted in Figure S10. Following the cooling process, the release of curcumin increased in all graft copolymers compared to the release measured at 37 °C, with a notable dependence on the composition. In the case of PNiPAAm-*g*-HbPG_4.3_, the cumulative drug release was increased by approximately 15% after 1 h of cooling. Similarly, in the case of PNiPAAm-*g*-HbPG_7.8_, an increase of approximately 20% was observed over the 2–3 h period. However, a substantial difference was not observed in the release profile of the highest HbPG-containing graft copolymer in the first hours, and the increase in the release was lower than 10% after 3 h following the temperature change. These observations were further supported by the kinetic evaluation of the curcumin release according to the Higuchi model (Figure S11), which revealed a predominantly diffusion-controlled release mechanism for all cases (*R* ^2^ > 0.94). The obtained Higuchi rate constants (*K*_H_) increase for all the graft copolymer-based nanoformulations when the temperature is reduced from 37 to 32 °C, indicating an acceleration of the curcumin release upon cooling. Specifically, the *K*_H_ values are 37.2, 16.6, and 10.6% h^–1/2^ at 37 °C and 39.0, 22.5, and 13.0% h^–1/2^ at 32 °C for the PNiPAAm-*g*-HbPG graft copolymer nanocarriers with increasing HbPG grafting density, respectively. Moreover, the amount of drug released after 24 h increased upon cooling, rising from 90.5% to 93.6%, 44.6% to 55.1%, and 27.6% to 33.6% with increasing HbPG content. While the released drug fraction for the PNiPAAm-*g*-HbPG_4.3_ sample is close to complete, the other two formulations still retain a portion of curcumin. These findings indicate that both the drug uptake and the release of the loaded drug can be induced by changing the temperature. In brief, the release of curcumin from the PNiPAAm-*g*-HbPG-based nanoformulations is sustained and continuous, and its rate can be decreased by increasing the HbPG content. Conversely, a lower HbPG content enables triggering of the release by decreasing the temperature, which induces the disaggregation of the nanoformulation, as illustrated in Figure 4D. As a result, the PNiPAAm-*g*-HbPG graft copolymers represent promising candidates for temperature-sensitive drug carrier and release systems.

### Colloidal Stability of the PNiPAAm-*g*-HbPG Graft Copolymers and the Curcumin-Loaded Formulations

The colloidal stability of drug-loaded polymeric nanoparticles at the temperature of administration in the body is crucial for medical applications. To further assess their behavior under physiologically relevant conditions, the colloidal stability of the PNiPAAm-*g*-HbPG graft copolymers, the PNiPAAm homopolymer, and curcumin in its free and nanoformulated forms was analyzed using turbidimetry.

As shown in Figure 5, increasing the temperature results in the formation of highly turbid systems above their CSTs. In contrast to the PNiPAAm, for which rapid aggregation results in macroscopic precipitation and sedimentation within approximately 100 min, the turbidity of the HbPG-grafted copolymers remains unchanged over the course of the measurement (see also Figure S12). These findings indicate that the stable nanoassemblies formed above the CSTs possess a hydrophobic central core coated with a HbPG-enriched hydrophilic shell, which ensures steric stabilization of the colloidal dispersion, as illustrated in Figure 5B. As illustrated in Figure 5A, free curcumin underwent rapid aggregation and sedimentation as indicated by the sharply increasing transmittance, highlighting the challenges associated with hydrophobic drugs, particularly their limited bioavailability. These processes can be slightly delayed by the presence of the PNiPAAm homopolymer, but the sedimentation remains fully complete at a rate very similar to that of the pure homopolymer. In contrast, the curcumin-containing PNiPAAm-*g*-HbPG copolymer dispersions, which form aggregates with mean diameters of 250–320 nm (Figure S13) and relatively narrow dispersity (PDI 0.05–0.07), demonstrate high and nearly constant turbidity over the investigated period. This finding indicates that the stability of the PNiPAAm-*g*-HbPG nanoaggregates predominantly governed by steric hindrance from the hydrophilic HbPG shell, with negligible electrostatic effects, i.e., HbPG as a part of the outer shell of the thermally formed nanoparticles can hinder further aggregation and sedimentation, as depicted schematically in Figure 5B.

**Figure 5:**
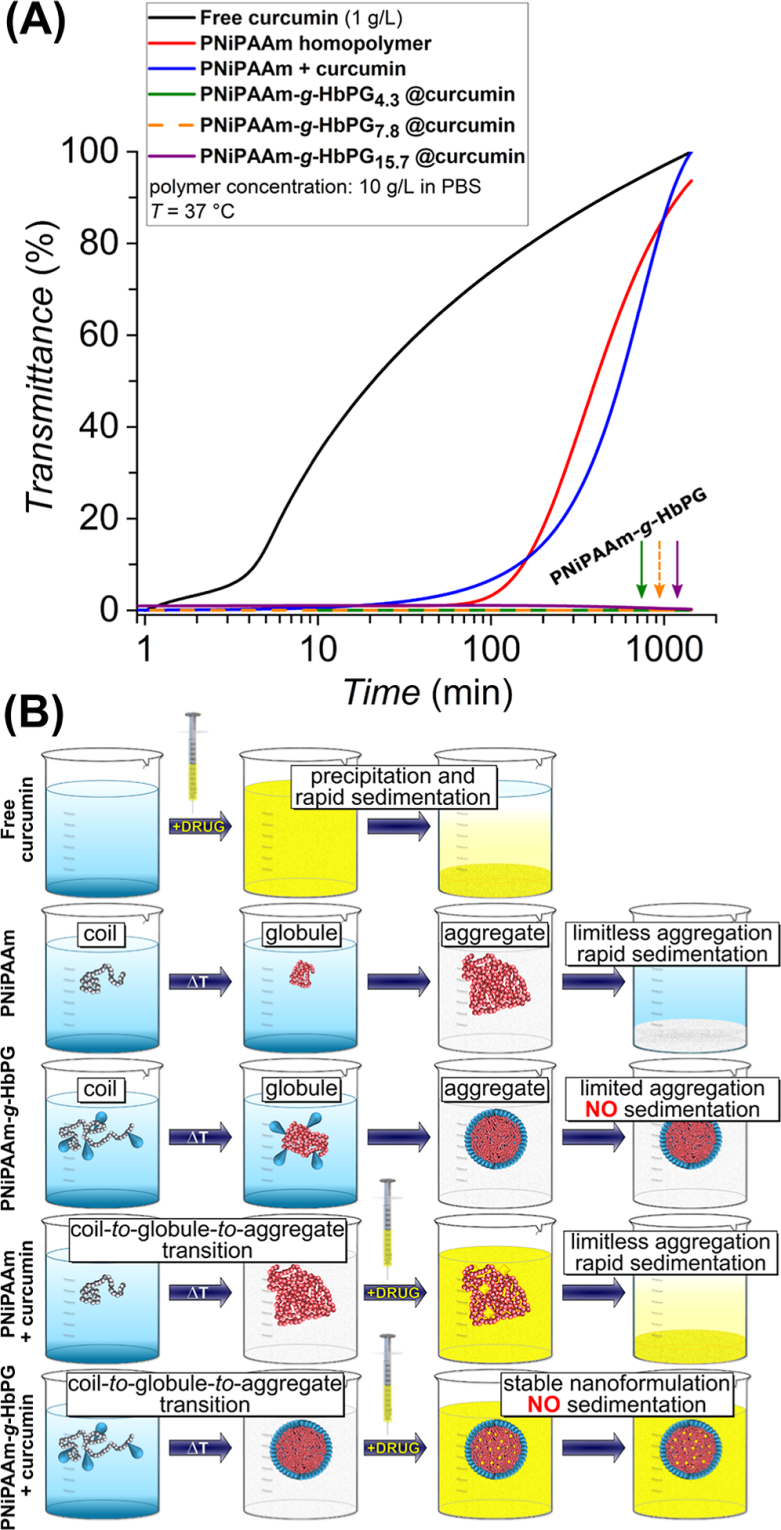
Transmittance of free curcumin, the PNiPAAm homopolymer, and the curcumin-loaded HbPG-grafted copolymer nanoaggregates as a function of time under physiologically relevant conditions (PBS, 37 °C) (A). Schematic illustration of the colloidal stability of the PNiPAAm homopolymer, the PNiPAAm-*g*-HbPG copolymer, and curcumin in free and encapsulated forms by the PNiPAAm homopolymer and the PNiPAAm-*g*-HbPG graft copolymers (B). (The numbers in sample codes indicate the HbPG contents in mol %.)

### *In Vitro* Susceptibility and Bioapplicability of the PNiPAAm-*g*-HbPG Graft Copolymers

To ascertain the suitability of the prepared PNiPAAm-*g*-HbPG graft copolymers as drug carriers in biological systems, their hemolytic activity and cytotoxicity were evaluated by using human red blood cells (RBC) and the HT-29 human cell line with epithelial morphology, respectively. It was found that none of the polymers tested impaired cell viability, even at the highest tested concentration of 2 or 2.5 g/L (Figure 6A,B). These findings suggest sufficient *in vitro* susceptibility of the PNiPAAm-*g*-HbPG graft copolymers, which is in line with our observations with PDEAAm-*g*-HbPG.^85^ Subsequently, the biological applicability of the PNiPAAm-*g*-HbPG graft copolymers as a drug delivery system was investigated by examining the cellular uptake and cytostatic effect of curcumin-loaded copolymer formulations on the HT-29 human colorectal adenocarcinoma cell line, which is frequently utilized to identify novel chemotherapeutic agents and more efficient anticancer treatments.^103,104^ Numerous studies in the past decade have demonstrated that curcumin possesses both antiproliferative and proapoptotic activities toward several human cancer cell lines, including those derived from colon cancer.^105^ As illustrated in Figure 6C, curcumin exhibits a considerable cytostatic effect of approximately 60% when administered in its free form at a concentration of 7 μM. Furthermore, similar to the case of PDEAAm-*g*-HbPG,^85^ the cytostatic effect is notably enhanced by utilizing PNiPAAm-*g*-HbPG graft copolymers for formulation. The improved anticancer activity can be explained by the results of the cellular uptake measurements (Figure 6D). In the case of the curcumin-loaded graft copolymers, the mean fluorescence intensity is 2-fold that of the unformulated curcumin, indicating that the graft copolymers significantly enhance the drug uptake of curcumin by cancer cells. These results provide clear evidence of the advantageous applicability of the HbPG-grafted PNiPAAm copolymers as drug delivery and release systems.

**Figure 6:**
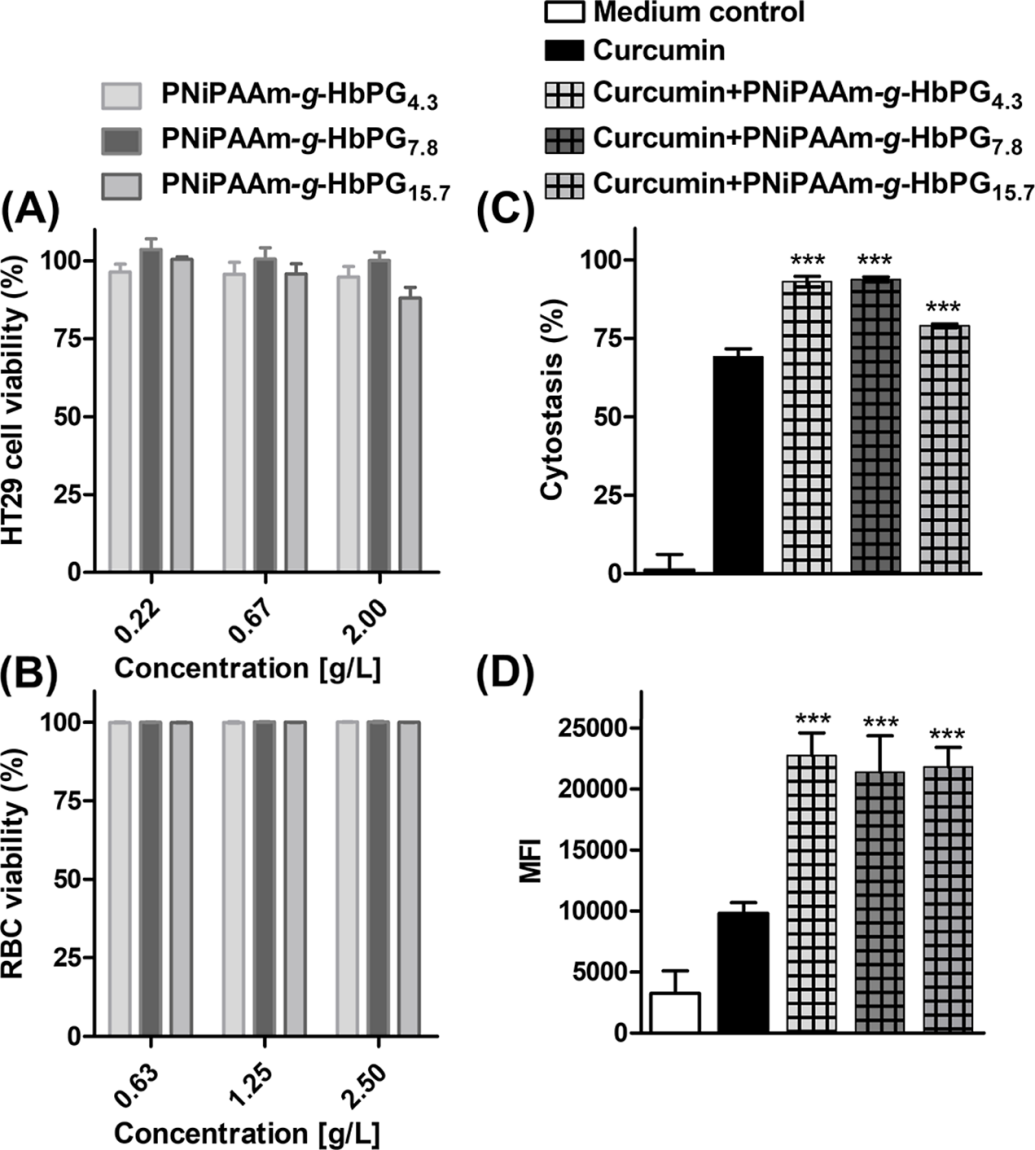
Biological evaluation of the PNiPAAm-*g*-HbPG graft copolymers and their curcumin-loaded analogs. (A) The percentage of cell viability caused by the polymers at the given concentrations, compared to medium-treated control cells, was measured on HT-29 colorectal cancer cells after 24 h of treatment at 37 °C (data represent mean ± Sd, *N* = 4). (B) The percentage of intact human RBC, compared to medium-treated negative control and a bee venom Melittin-treated positive control (data are presented as mean ± Sd, *N* = 3). (C) The cytostatic effect of curcumin and curcumin-loaded graft copolymers on HT-29 cells (bars represent mean ± Sd, *N* = 4). (D) Internalization of curcumin and its PNiPAAm-*g*-HbPG-encapsulated formulations in HT-29 cells expressed by mean fluorescence intensity (MFI) (bars represent mean ± Sd, *N* = 3). Statistical analysis was performed using one-way analysis of variance (ANOVA), followed by Tukey’s post hoc test, comparing curcumin vs curcumin-loaded graft copolymers, statistical significance: ***p < 0.001. (The numbers in sample codes indicate the HbPG contents in mol %.)

## Conclusions

In this study, HbPG-grafted poly­(*N*-isopropylacrylamide) (PNiPAAm-*g*HbPG) thermoresponsive copolymers with varying grafting density were successfully synthesized through the grafting of monofunctional amino-HbPG onto reactive *N*-hydroxyl succinic amide active ester-containing functional PNiPAAm copolymers. The thermoresponsive behavior of the copolymers was investigated using turbidimetry and DLS measurements, which demonstrated that the CST of the PNiPAAm-*g*-HbPG copolymers increases with increasing HbPG content at a given polymer concentration, conversely to the P­(NiPAAm-*co*-NAOS) copolymer precursors, where a decrease in CST was observed with increasing hydrophobic NAOS content. The reversible aggregation–disaggregation transition of the PNiPAAm-*g*-HbPG graft copolymers was demonstrated by a heating/cooling cycle between the small, coiled particles (∼5 nm) at room temperature and larger copolymer aggregates with a 300–500 nm mean diameter above the CST.

The encapsulation experiments confirmed that curcumin was effectively loaded into the nanoscale aggregates formed by the graft copolymers. It was found that the encapsulated curcumin concentration increases with increasing polymer concentration up to 2–3 orders of magnitude higher than that of the water solubility of curcumin. An increase in temperature or a reduction in HbPG content in the PNiPAAm-*g*-HbPG graft copolymers enhanced the curcumin loading, which can be attributed to the increased hydrophobic character of the PNiPAAm polymer backbone. Another significant consequence of HbPG grafting onto PNiPAAm is the sustained release of curcumin from the drug formulations. The extent of sustained drug release was found to be lower in graft copolymers with higher HbPG content, resulting in a denser and more hydrated surface region of the polymer nanoparticles, which may hinder the release of hydrophobic curcumin to some extent. Temperature variations can also affect the release profile of curcumin from the graft copolymers; i.e., lowering the release temperature by 5 °C results in a higher release rate, especially for copolymers with lower grafting density. This enables site-specific drug release induced by localized cooling. Due to the hydrophilic branched grafted side chain, exceptional colloidal stability of the PNiPAAm-*g*-HbPG copolymers and the drug-loaded formulations were obtained under physiologically relevant conditions, suggesting that the formed nanoassemblies have a stabilizing outer shell enriched with the hydrophilic HbPG side chains.

The graft copolymers were found to be nontoxic to epithelial cells and did not cause hemolysis of human RBC even at relatively high concentrations, i.e., 2 and 2.5 g/L, thereby indicating their *in vitro* susceptibility as an essential requirement. The bioactivity study on the HT-29 colon cancer cell line shows that the nanoformulation of curcumin by the graft copolymers enhances the internalization rate, resulting in a significantly higher anticancer activity for the curcumin-loaded copolymer nanoparticles than that of free curcumin. The tested cell line can effectively model other colorectal cancer cell lines, such as HC-T15, HCT-116, or SW-620, and this cell line is also often used as a model system for studying multiple aspects of cancer biology, including cytostasis, cell proliferation, differentiation, and drug resistance. In particular, the HT-29 cell line is used in numerous protocols involving multicellular intestinal systems and organoids,^108^ and HT-29 xenograft mouse model (subcutaneous and metastatic) is also available,^109^ enabling preclinical therapeutic studies. Considering all of these aspects, our results show promising translational potential. Overall, the PNiPAAm-*g*-HbPG graft copolymers exhibit several favorable characteristics, including tunable thermoresponsiveness, excellent colloidal stability, and *in vitro* susceptibility. Due to the thermally triggered encapsulation and sustained drug release properties with enhanced bioactivity, the PNiPAAm-*g*-HbPG graft copolymers have significant potential as advanced drug delivery and release systems.

## Supplementary Material



## Notes

Cloud point (*T*_CP_) and clearing point (*T*_CL_) temperatures of the PNiPAAm homopolymer, the P­(NiPAAm-*co*-NAOS) copolymers, and the PNiPAAm-*g*-HbPG copolymers determined by turbidimetry measurements (Table S1); ^1^H NMR spectra of amine-monofunctional HbPG and P­(NiPAAm-*co*-NAOS) copolymer precursors and that of the PNiPAAm-*g*-HbPG graft copolymers (Figure S1); the GPC chromatograms, molecular weight distribution curves and ^1^H NMR spectra of the phthalimide-monofunctional HbPG precursor and the amine-monofunctional HbPG (Figures S2–S4); the GPC chromatograms of NH_2_–HbPG, P­(NiPAAm-*co*-NAOS), and PNiPAAm-*g*-HbPG copolymers (Figure S5); the first derivative of the transmittance–temperature curves of the copolymers (Figures S6 and S7); transmittance vs temperature curves and the first derivative of the transmittance–temperature curves of the PNiPAAm homopolymer (Figure S8); decrease of curcumin concentration and cumulative release of the encapsulated curcumin as a function of time for the PNiPAAm-*g*-HbPG-grafted copolymers and free curcumin (Figures S9 and S10); the cumulative curcumin release as a function of the square root of time (Figure S11); transmittance of the PNiPAAm homopolymer and the HbPG-grafted copolymers as a function of time under physiologically relevant conditions (Figure S12); the size distribution curves of the curcumin-loaded PNiPAAm-*g*-HbPG graft copolymers (Figure S13); UV–vis spectra of the curcumin and PNiPAAm-*g*-HbPG copolymers (Figures S14 and S15); and absorbance values of curcumin solutions in ethanol as a function of curcumin concentration (Figure S16) (PDF)
