# Supplementary material for: Thermoresponsive Graft Copolymers of N‑Isopropylacrylamide and Hyperbranched Polyglycerol as Thermally Induced Drug Delivery and Release Nanoformulation Systems for Curcumin with High Colloidal Stability and Enhanced Anticancer Effect
Source: ACS Omega. 2026 Jan 8;11(3):3780–95. doi: 10.1021/acsomega.5c05838 (PMC12854384; doi:10.1021/acsomega.5c05838)
Supplement: Supplementary file 1 [file ao5c05838_si_001.pdf]

Supporting Information

Thermoresponsive Graft Copolymers of *N*-Isopropylacrylamide and Hyperbranched Polyglycerol as Thermally Induced Drug Delivery and Release Nanoformulation Systems for Curcumin with High Colloidal Stability and Enhanced Anticancer Effect

György Kasza[†,*], Ákos Fábián[†], Dóra Fecske[†,‡], Anna Petróczy[†,‡], Kata Horváti[§],

and Béla Iván[†,*]

[†]Polymer Chemistry and Physics Research Group, Institute of Materials and Environmental Chemistry, HUN-REN Research Centre for Natural Sciences, Magyar tudósok körútja 2, H-1117 Budapest, Hungary

[‡]Hevesy György Doctoral School of Chemistry, ELTE Eötvös Loránd University, Pázmány Péter sétány 1/A, H-1117 Budapest, Hungary

[§]MTA–HUN-REN "Momentum" Peptide-Based Vaccines Research Group, Institute of Materials and Environmental Chemistry, HUN-REN Research Centre for Natural Sciences, Magyar tudósok körútja 2, H-1117 Budapest, Hungary

*Corresponding author: kasza.gyorgy@ttk.hu (Gy.K.); ivan.bela@ttk.hu (B.I.)

Supplementary Tables

Table S1. The cloud point (T_{CP}) and clearing point (T_{CL}) temperatures of the PNiPAAm homopolymer, the P(NiPAAm-*co*-NAOS) copolymers, and the PNiPAAm-*g*-HbPG copolymers determined by turbidimetry measurements.

Samples	in water						in PBS					
	1 g/L		5 g/L		10 g/L		1 g/L		5 g/L		10 g/L	
	T_{CP} °C	T_{CL} °C	T_{CP} °C	T_{CL} °C	T_{CP} °C	T_{CL} °C	T_{CP} °C	T_{CL} °C	T_{CP} °C	T_{CL} °C	T_{CP} °C	T_{CL} °C
P(NiPAAm-*co*-NAOS)$_{4.3}$	34.0	30.4	-	-	-	-	-	-	-	-	-	-
P(NiPAAm-*co*-NAOS)$_{7.8}$	32.0	28.2	-	-	-	-	-	-	-	-	-	-
P(NiPAAm-*co*-NAOS)$_{15.7}$	27.7	23.9	-	-	-	-	-	-	-	-	-	-
PNiPAAm	35.4	32.0	33.1	30.6	32.1	28.6	33.0	29.0	31.8	28.2	30.6	27.6
PNiPAAm-*g*-HbPG$_{4.3}$	41.1	39.0	35.4	33.6	33.1	30.9	38.3	33.8	33.5	30.7	31.3	28.5
PNiPAAm-*g*-HbPG$_{7.8}$	43.9	41.2	37.6	35.8	35.6	32.7	43.7	38.6	35.0	32.4	34.2	30.2
PNiPAAm-*g*-HbPG$_{15.7}$	53.5	51.6	42.1	38.3	40.8	37.2	48.3	47.2	39.4	36.9	37.4	32.9

Supplementary Figures

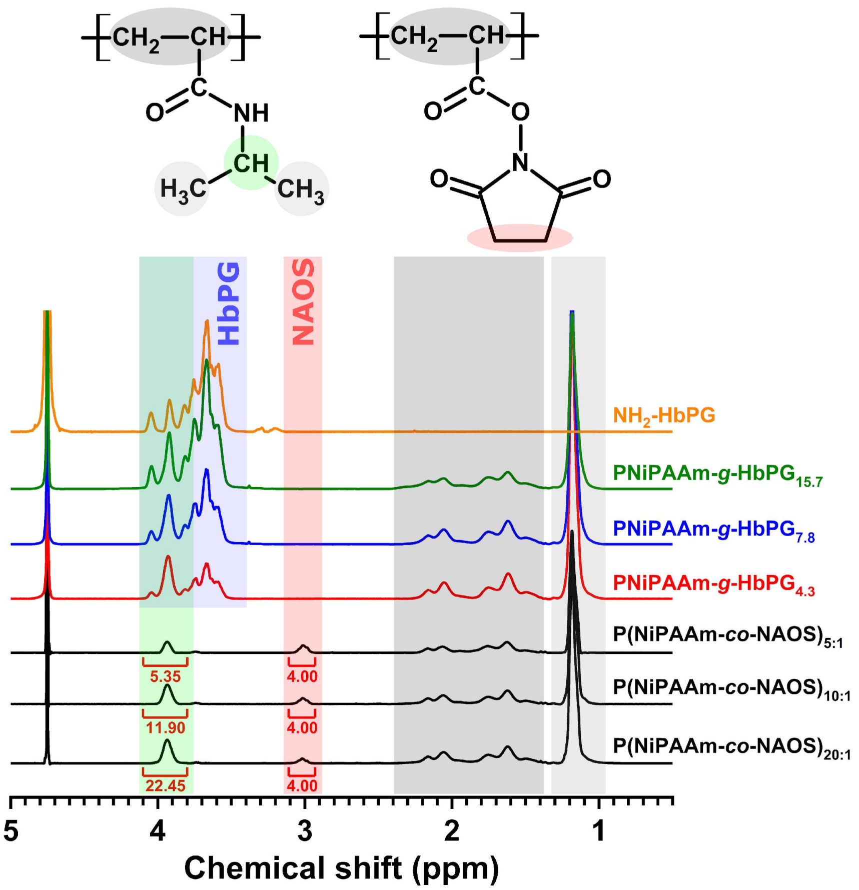

Figure S1. ^{1}H NMR spectra of amine-monofunctional HbPG and P(NiPAAm-*co*-NAOS) copolymer precursors and that of the PNiPAAm-*g*-HbPG graft copolymers.

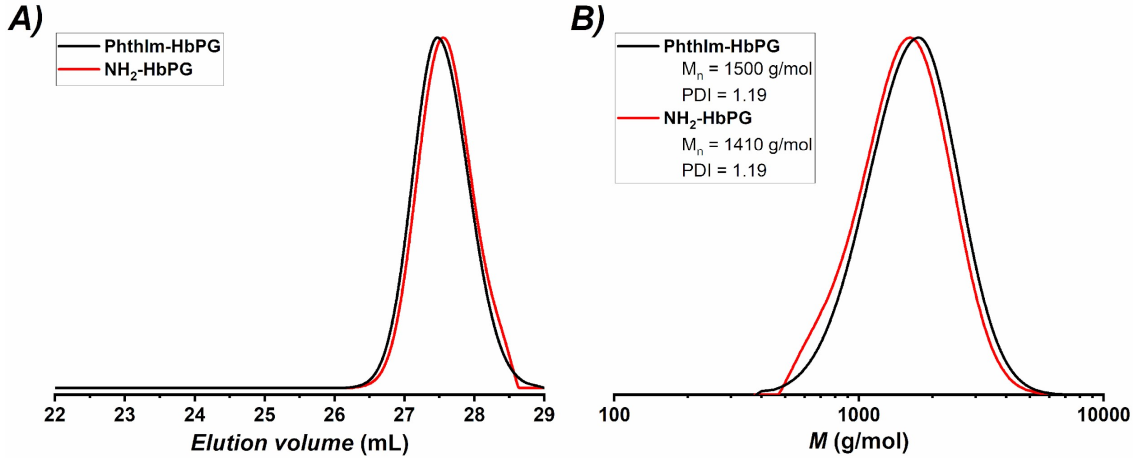

Figure S2. The GPC chromatograms (A) and molecular weight distribution curves (B) of the phthalimide- (PhthIm-) and amine-monofunctional HbPG (NH_2-HbPG).

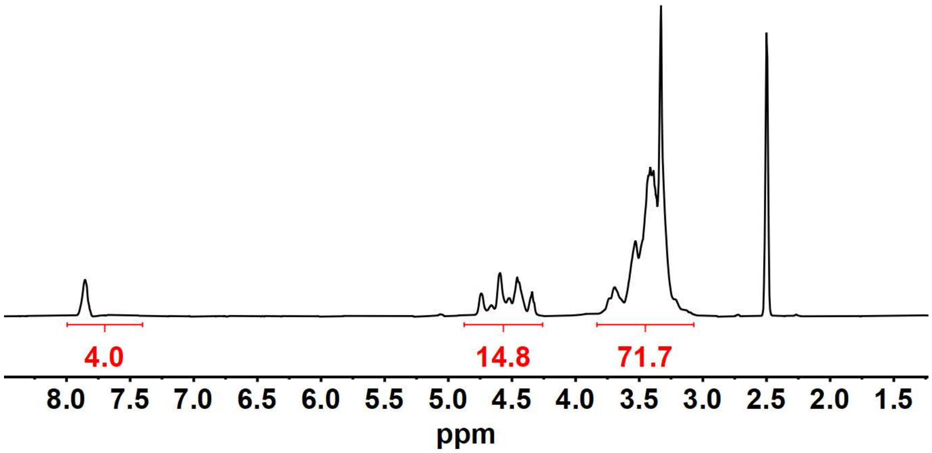

Figure S3. The ^{1}H NMR spectrum of the phthalimide-monofunctional HbPG precursor in DMSO-d_6.

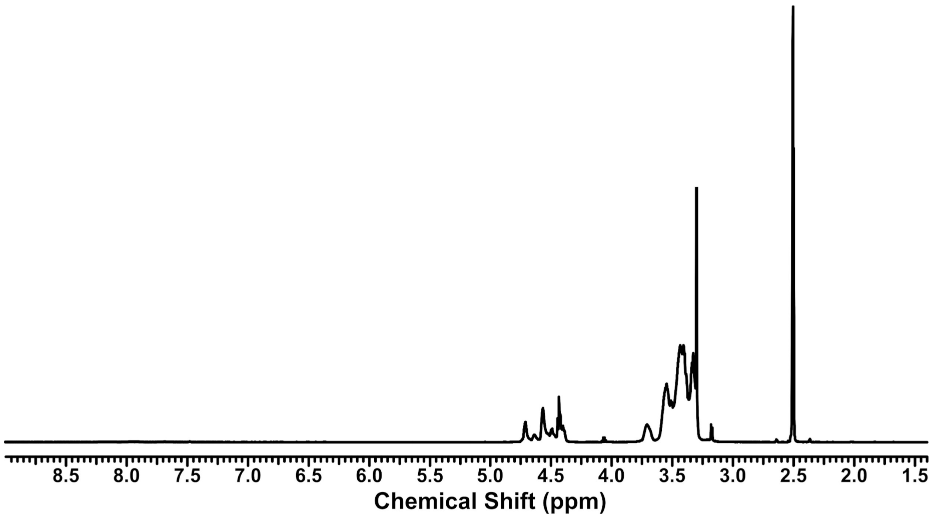

Figure S4. The ^{1}H NMR spectrum of the amine-monofunctional HbPG in DMSO-d_6.

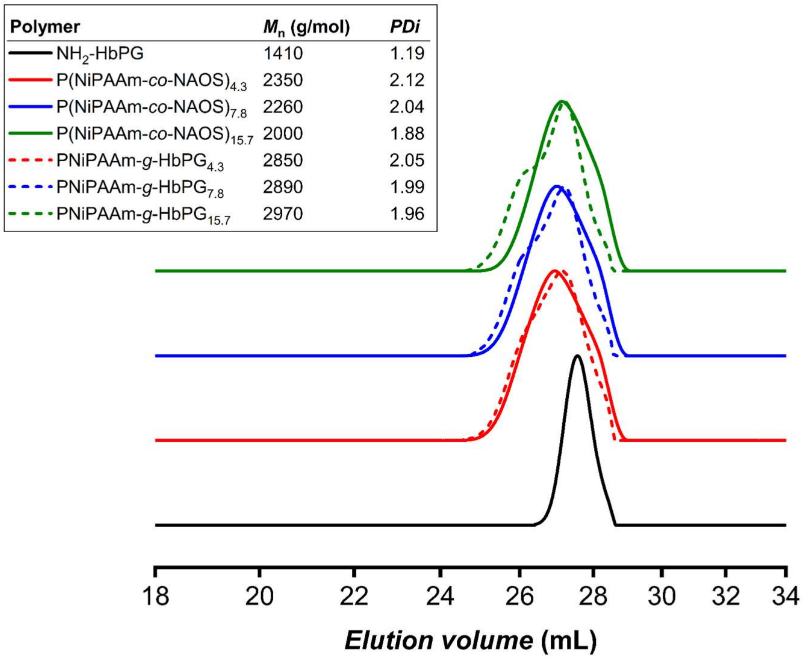

Figure S5. The GPC chromatograms of NH_2-HbPG (black line), P(NiPAAm-*co*-NAOS) (solid lines) and PNiPAAm-*g*-HbPG (dashed lines) copolymers. (The numbers in sample codes indicate the comonomer contents in mol%.)

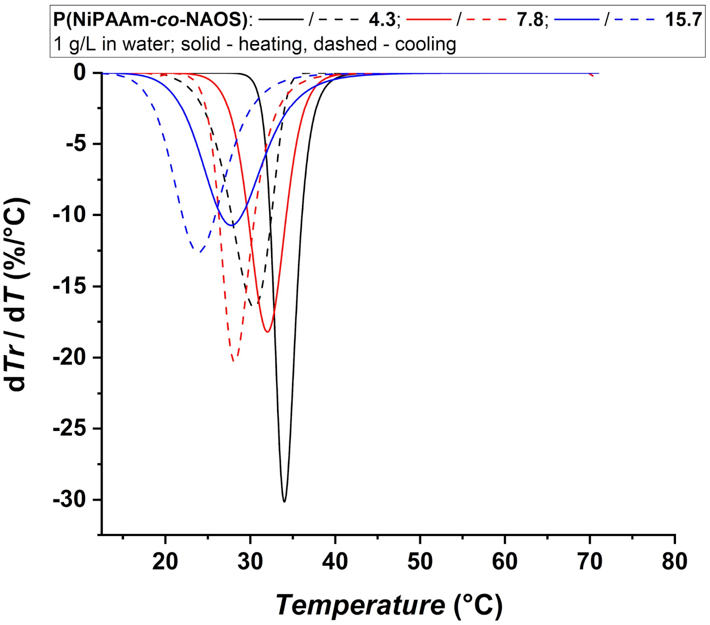

Figure S6. The first derivative of the transmittance-temperature curves of the P(NiPAAm-*co*-NAOS) copolymers at a concentration of 1 g/L in water. (The numbers in sample codes indicate the comonomer contents in mol%.)

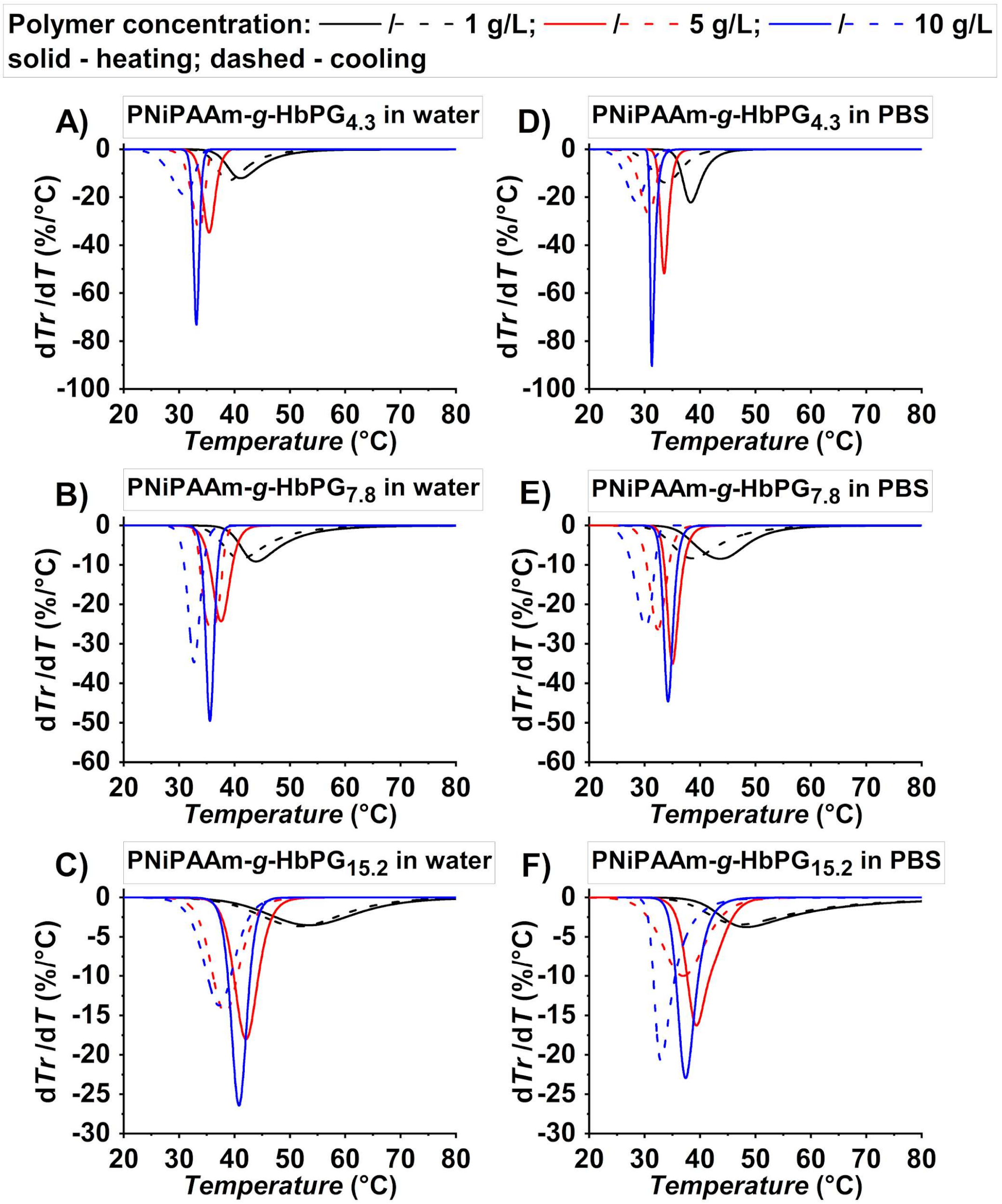

Figure S7. The first derivative of the transmittance-temperature curves of the PNiPAAm-*g*-HbPG copolymers at different concentrations (1, 5, 10 g/L) in water (A,B,C) and in PBS (D,E,F). (The numbers in sample codes indicate the comonomer contents in mol%.)

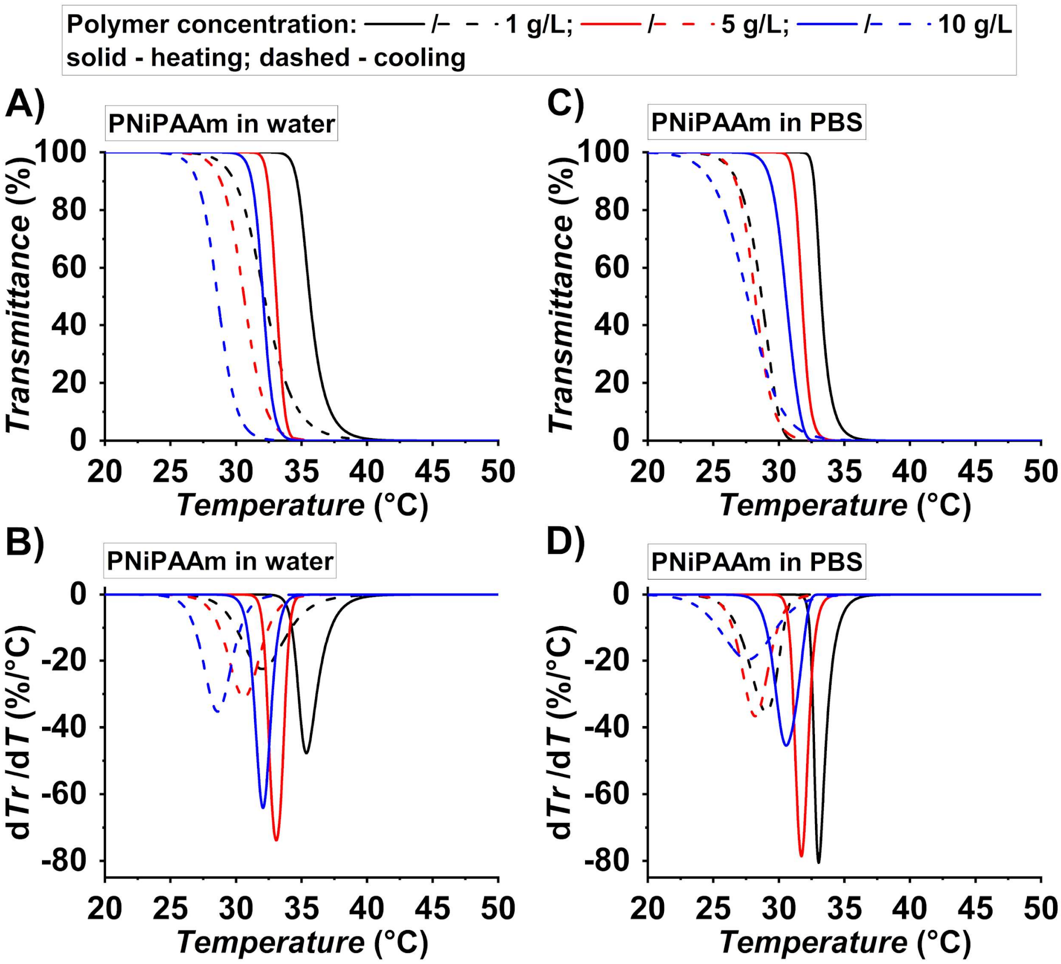

Figure S8. Transmittance vs. temperature curves (A,C) and the first derivative of the transmittance-temperature curves (B,D) of the PNiPAAm homopolymer at different concentrations (1, 5, 10 g/L) in water (A,B) and in PBS (C,D).

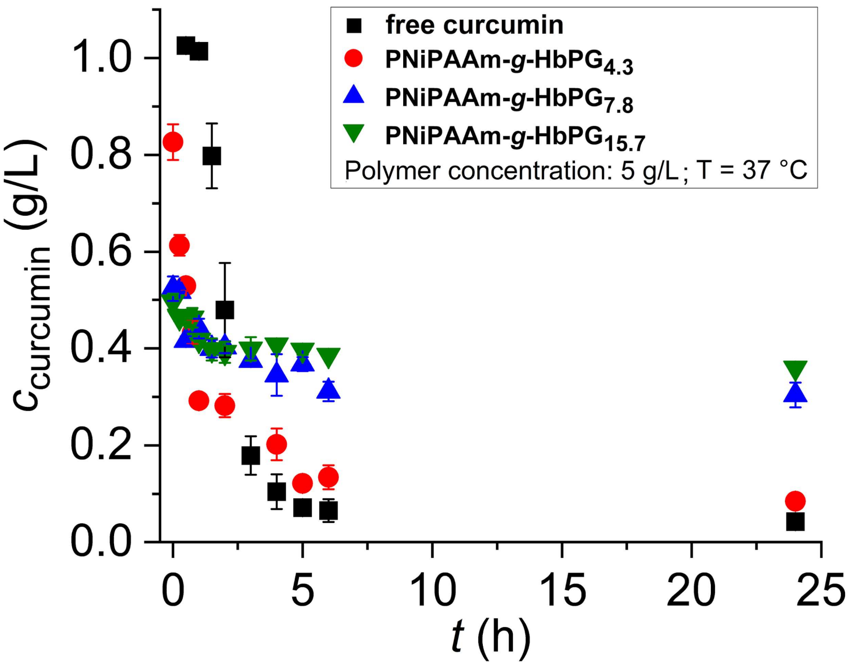

Figure S9. Decrease of curcumin concentration as a function of time for the PNiPAAm-*g*-HbPG grafted copolymers and free curcumin. Encapsulation of curcumin was performed at 37 °C. (The numbers in sample codes indicate the comonomer contents in mol%.)

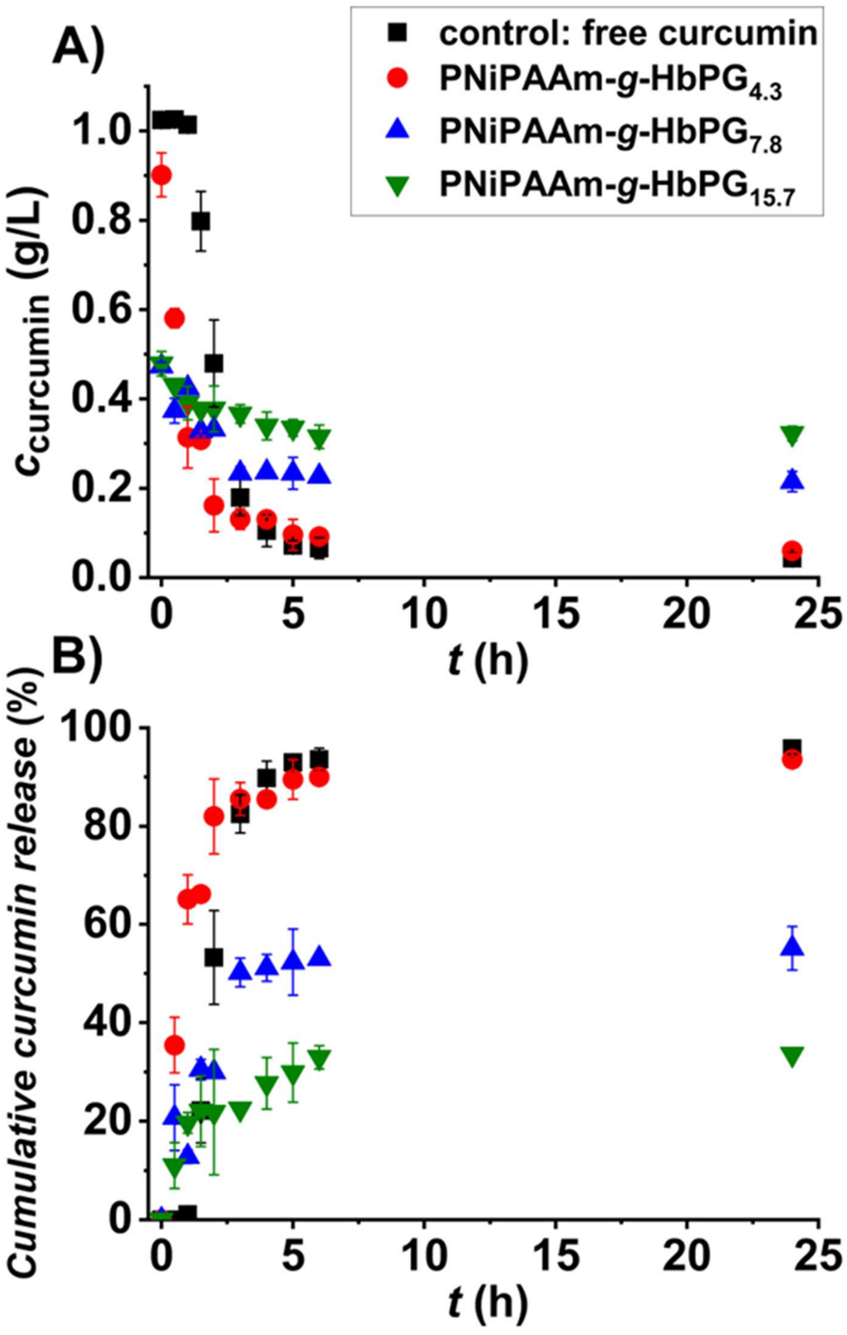

Figure S10. Decrease of curcumin concentration as a function of time for the PNiPAAm-*g*-HbPG grafted copolymers and free curcumin (A). Cumulative release of the encapsulated curcumin as a function of time for the PNiPAAm-*g*-HbPG graft copolymers and free curcumin as a control sample (B). The temperature of the release medium was decreased from 37 °C to 32 °C after an hour. (The numbers in sample codes indicate the comonomer contents in mol%.)

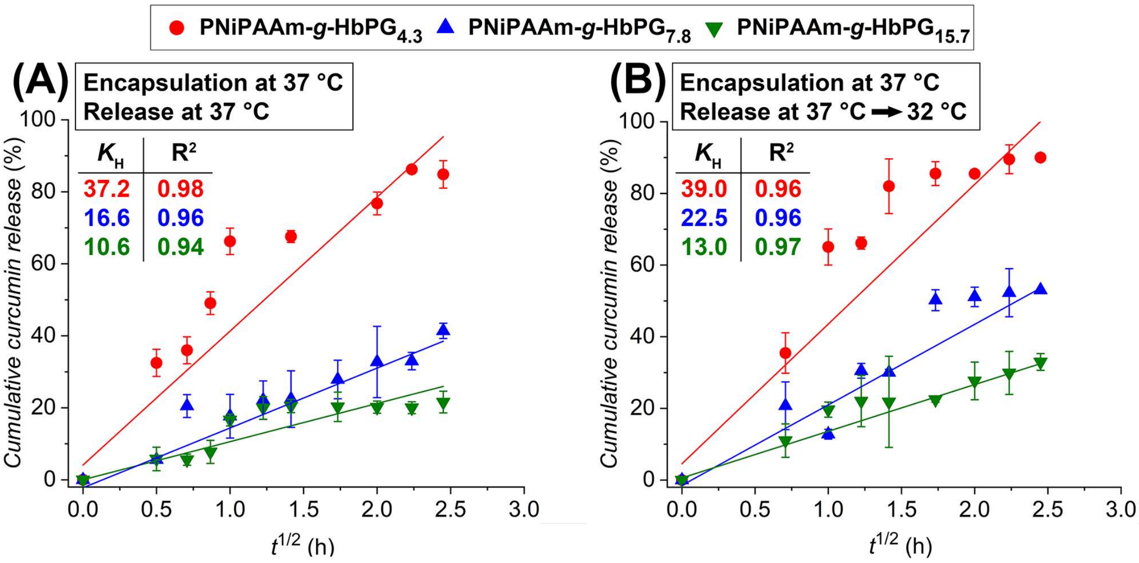

Figure S11. The cumulative curcumin release from the PNiPAAm-*g*-HbPG graft copolymer nanoformulations as a function of the square root of time. The temperature of the release medium was 37 °C (**A**) and decreased from 37 °C to 32 °C after an hour (**B**).

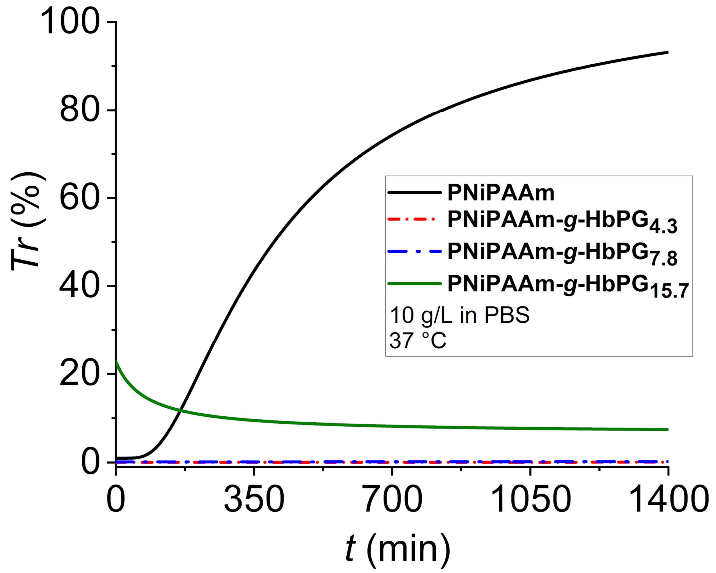

Figure S12. Transmittance of the PNiPAAm homopolymer and the HbPG-grafted copolymers as a function of time under physiologically relevant conditions (PBS, 37 °C). (The numbers in sample codes indicate the comonomer contents in mol%.)

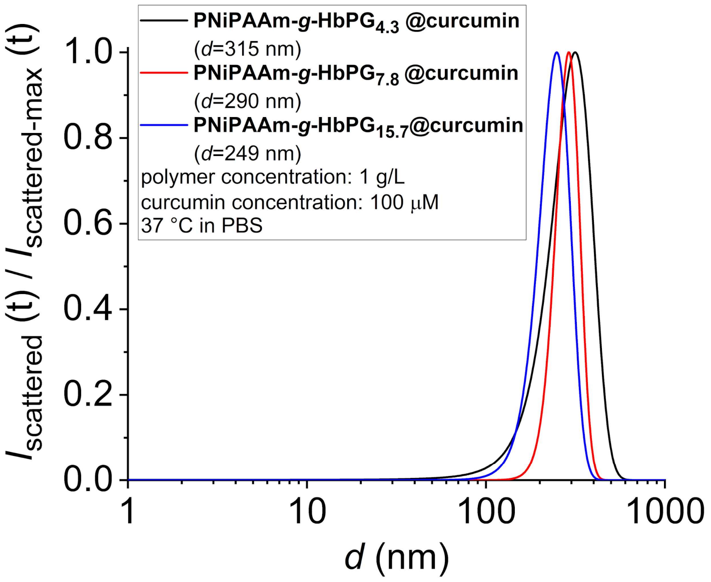

Figure S13. The size distribution curves of the curcumin-loaded PNiPAAm-*g*-HbPG graft copolymers at 37 °C in PBS determined by DLS measurements. (The numbers in sample codes indicate the comonomer contents in mol%.)

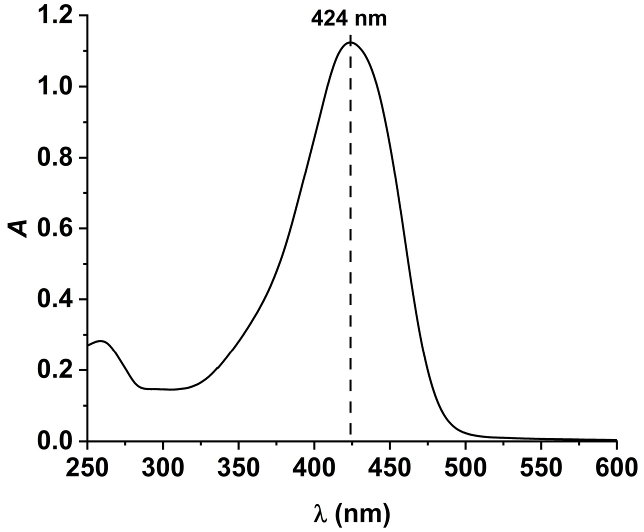

Figure S14. The UV-Vis spectrum of curcumin in absolute ethanol (concentration: 18 µg/mL). The dashed line indicates the position of the absorbance maximum of curcumin.

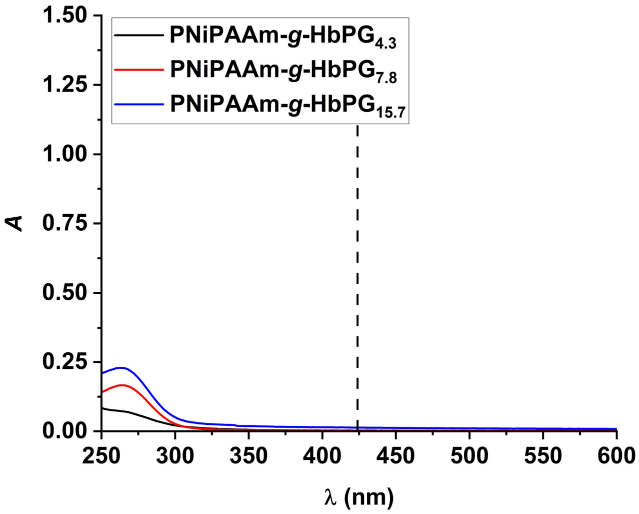

Figure S15. The UV-Vis spectra of the PNiPAAm-*g*-HbPG copolymers (polymer concentration: 10 g/L). The dashed line indicates the position of the absorbance maximum of curcumin.

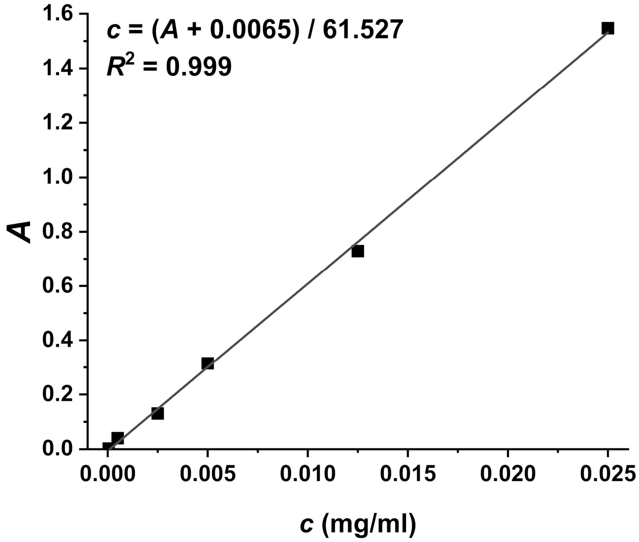

Figure S16. Absorbance values of curcumin solutions in ethanol at 424 nm as a function of curcumin concentration, and the determined equation of the calibration curve.
